# Identification of a 31-bp Deletion in the *RELN* Gene Causing Lissencephaly with Cerebellar Hypoplasia in Sheep

**DOI:** 10.1371/journal.pone.0081072

**Published:** 2013-11-19

**Authors:** Aroa Suárez-Vega, Beatriz Gutiérrez-Gil, Inmaculada Cuchillo-Ibáñez, Javier Sáez-Valero, Valentín Pérez, Elsa García-Gámez, Julio Benavides, Juan Jose Arranz

**Affiliations:** 1 Dpto. Producción Animal, Universidad de León, León, León, Spain; 2 Instituto de Neurociencias de Alicante, Universidad Miguel Hernández, CSIC, Sant Joan d’Alacant, Alicante, Spain; 3 Centro de Investigación Biomédica en Red sobre Enfermedades Neurodegenerativas (CIBERNED), Universidad Miguel Hernández, Sant Joan d’Alacant, Alicante, Spain; 4 Dpto. de Sanidad Animal, Universidad de León, León, León, Spain; 5 Instituto de Ganadería de Montaña, Universidad de León CSIC, Grulleros, León, Spain; The University of Melbourne, United States of America

## Abstract

Lissencephaly is an inherited developmental disorder in which neuronal migration is impaired. A type of lissencephaly associated with cerebellar hypoplasia (LCH) was diagnosed in a commercial flock of Spanish Churra sheep. The genotyping of 7 affected animals and 33 controls with the OvineSNP50 BeadChip enabled the localization of the causative mutation for ovine LCH to a 4.8-Mb interval on sheep chromosome 4 using genome-wide association and homozygosity mapping. The *RELN* gene, which is located within this interval, was considered a strong positional and functional candidate because it plays critical roles in neuronal migration and layer formation. By performing a sequencing analysis of this gene’s specific mRNA in a control lamb, we obtained the complete CDS of the ovine *RELN* gene. The cDNA sequence from an LCH-affected lamb revealed a deletion of 31 bp (c.5410_5440del) in predicted exon 36 of *RELN*, resulting in a premature termination codon. A functional analysis of this mutation revealed decreased levels of *RELN* mRNA and a lack of reelin protein in the brain cortex and blood of affected lambs. This mutation showed a complete concordance with the Mendelian recessive pattern of inheritance observed for the disease. The identification of the causal mutation of LCH in Churra sheep will facilitate the implementation of gene-assisted selection to detect heterozygous mutants, which will help breeders avoid at-risk matings in their flocks. Moreover, the identification of this naturally occurring *RELN* mutation provides an opportunity to use Churra sheep as a genetically characterized large animal model for the study of reelin functions in the developing and mature brain.

## Introduction

Lissencephaly (LIS), which literally means “smooth brain”, refers to a group of rare malformations that share a common feature: absent or abnormal brain convolutions caused by the aberrant migration of postmitotic neurons to the developing cortex. Although different forms of LIS have been described, there is still no final consensus on their classification. The classification system proposed by Jissendi-Tchofo et al. [1] divides this disease into four different groups: classic lissencephaly (cLIS), variant lissencephaly (vLIS), cobblestone complex, and related muscular dystrophy syndrome. 

Lissencephalies are a genetically heterogeneous group of disorders. There are seven different human LIS-related phenotypes described in the Online Mendelian Inheritance in Man (OMIM) database (#607432, #300067, #300215, #257320, #611603, #614019 and #615191). These phenotypes have been linked to different aberrant proteins related to the cytoskeleton of neural cells (Platelet-activating factor acetylhydrolase IB subunit alpha (PAFAH1B1 or LIS1), Doublecortin (DCX) , Tubulin alpha-1A (TUBA1A)), signaling molecules (Reelin (RELN), Very-low-density lipoprotein receptor (VLDLR)), molecules that modulate stop signals for migrating neurons (Protein-O-mannosyltransferase 1 (POMT1), Protein O-linked-mannose beta-1,2-N-acetylglucosaminyltransferase 1 (POMGnT1), Fukutin (FKTN)) and other factors shown to modulate neuronal migration (Aristaless related homeobox (ARX), Laminin alpha 1 (LAMA1)) [2].

Lissencephaly with cerebellar hypoplasia (LCH) is a type of LIS included within the vLIS group [1]. In this form of LIS, the thickened cortex is associated with significant cerebellar underdevelopment. Six subtypes of LCH have been described [3]. Several genes that are involved in the gestational migration of neurons have been linked to human LCH: *PAFAH1B1* [3], *DCX* [3], *RELN* [4], *VLDLR* [1,5] and *TUBA1A* [6].

Although rarely observed clinically, inherited forms of LCH are well known in humans and mouse mutant models like the *reeler* phenotype [7] due to its importance in understanding brain development. However, beyond its known forms in rodents, LCH is rarely described in veterinary medicine. Two litters of Wire Fox Terriers and Irish Setters [8] and one two-year-old cat [9] represent the only cases of LCH described in domestic animals, to our knowledge.

Between 2004 and 2012, an inherited form of LCH was identified in a commercial flock of Spanish Churra sheep. The affected lambs exhibited severe ataxia, were unable to stand by themselves and died several days after birth. Pathological examination showed alterations in the brain characterized by agyria, pachygyria and cerebellar hypoplasia. The segregation of this disease in the affected pedigrees was consistent with a recessive mode of Mendelian inheritance [10].

Recently, high-density single-nucleotide polymorphism (SNP) arrays have provided an opportunity to explore the genomes of livestock species to identify genes and mutations that cause inherited defects [11]. In sheep, the Illumina OvineSNP50 BeadChip has proven to be a useful tool for the identification of causal mutations underlying the genetic control of diseases with Mendelian inheritance patterns [12-14]. Using this genomic tool, we performed genome-wide association and homozygosity mapping analyses to map the ovine locus associated with the form of LCH identified in Churra sheep. These analyses allowed the identification of a strong positional and functional candidate gene, which was subjected to later analyses with the aim of identifying the causal mutation underlying the studied developmental malformation. 

Hence, the present study identified the causal mutation for LCH in sheep, which will allow the direct implementation of gene-assisted selection into breeding practices by enabling the detection of phenotypically normal carriers. By deciphering the genetic basis of this ovine disease, the current work also provides a potential large animal model for human LCH, the study of brain development and the development of possible gene therapy treatments.

## Materials and Methods

### Ethics statement

Blood and tissue samples were collected from rams, ewes and lambs by qualified veterinarians following standard procedures and conducted under license issued in accordance with European Union legislation (European Community Directive, 86/609/EC and Directive 2010/63/EU of the European Parliament and of the Council). All animals were managed in accordance with the guidelines for the accommodation and care of animals.

The DNA samples used in this study were extracted from blood leucocytes. For this purpose 4 mL of blood was obtained by jugular venepuncture. 

Tissues were collected immediately after euthanasia (performed by a qualified veterinarian with an intravenous injection of veterinary euthanasia drug (T-61, Intervet)). As the samples were from a commercial flock that underwent veterinary examination, we were in a special situation in veterinary medicine and there was no “animal experiment” according to the legal definitions in Spain (Animal care legislation “Ley 32/2007”). According to the Ethics Commission of the University of Leon, formal ethical approval is not required under these circumstances.

### Animals

Several LCH-affected animals were found in a commercial flock of Spanish Churra sheep belonging to the Spanish Churra sheep breeders’ association (ANCHE). For many years, the breeding strategy for the flock has been based on the use of rams from within this flock. Although a larger number of possible LCH cases had been detected in the flock, we examined seven LCH-affected animals, all of which exhibited the same clinical features at birth. All these animals could not stand by themselves and showed severe ataxia and muscular hypertonia. The affected lambs had difficulty suckling from their mothers and died a few days after birth. The post-mortem examination showed agyria with only a few rudimentary sulci and gyri, as well as marked cerebellar hypoplasia. The microscopically normal layering of the cerebral cortex was disorganized, and immunohistochemical staining of the neurofilaments revealed a three-layered cortex instead of the six layers that appear in normal brains. All these findings were consistent with LCH [10].

### Samples and pedigree information

Blood samples were collected from 63 animals from the LCH-affected flock. Seven of these animals were affected lambs. DNA was extracted from blood using the salting out procedure [15]. To confirm the familial relationships recorded in the flock register, all the samples were analyzed for a set of 19 microsatellite markers in a single multiplex PCR reaction [16]. To construct the pedigree of the sampled animals information about the ram parents, grandparents and great-grandparents was obtained from the ANCHE database.

The pedigree was constructed to confirm the monogenic autosomic mode of inheritance of this ovine disease reported [10] and to identify the common founder responsible of the establishment of the disease in this flock. For this purpose, we used CraneFoot software [17].

### Mapping the causative gene for lissencephaly

A total of 40 DNA samples were genotyped using the Illumina OvineSNP50 BeadChip. These samples included 20 unrelated, healthy Churra individuals from different flocks of the Churra Selection Nucleus and 20 animals from the affected flock. Seven of the sheep from the affected flock were LCH lambs, and the rest were related to them (sires, dams, siblings or half-siblings of the affected lambs). Raw data will be shared upon request.

The results were analyzed using PLINK software [18]. Firstly, a quality control procedure was performed to eliminate animals with call rates lower than 0.95 and SNPs with genotyping rate values lower than 0.05 and minor allele frequencies lower than 0.01. After the frequency and genotype pruning, 47,864 SNPs were considered in the subsequent analysis. The --*assoc* option of the PLINK software was used to perform a case-control genome-wide association (GWA) analysis. The empirical p-values were corrected by performing a permutation procedure implemented in PLINK with 100,000 permutations. 

Finally, an analysis of runs of homozygosity was carried out. The LCH cases were filtered to identify allele-sharing regions. The SNPs flanking the consensus region according to this analysis were used to locate this region in the Ovine Genome Assembly v3.1 browser (http://www.livestockgenomics.csiro.au/cgi-bin/gbrowse/oarv3.1/). Genes located within the homozygosity block were evaluated as putative positional candidate genes. A second analysis of runs of homozygosity was performed on only 10 unrelated healthy Churra sheep to confirm that these control individuals did not share any common homozygous regions with the affected lambs.

### Isolation of the complete coding sequence of the ovine RELN gene and mutation scanning

Because of its association with human LCH [4], the *RELN* gene, located within the homozygosity block (4.8 Mb), was identified as the most promising candidate. Due to the large size of the *RELN* gene, which encompasses 450 kb distributed across 65 exons in the human genome [19], we first examined the *RELN* coding sequence. Brain tissues from a control lamb and an affected lamb were collected for RNA extraction. The use of the animals was in compliance with the guidelines approved by the University of Leon Ethics Commission. The brain tissue samples were harvested immediately after euthanasia and were preserved in RNA Stabilization Reagent (RNAlater, Ambion). Slices of up to 500 mg of brain tissue were processed with the RNeasy Lipid Tissue Midi Kit (Qiagen) to extract total RNA.

For the primer design, due to the absence of a published RNA sequence for the ovine *RELN* gene, we first constructed a virtual mRNA sequence based on exons obtained by a BLAST comparison of the GenBank human (NM_005045) and mouse (NM_011261) mRNA sequences against the genomic *RELN* sequence obtained from the ovine genome assembly v3.1. For this procedure, we used the web-based Spidey software, available at http://www.ncbi.nlm.nih.gov/spidey/.

The virtual mRNA sequence thus generated was used to design 32 primer pairs to cover all the *RELN* coding sequence, using the Primer3 software [20]. The information from the human and mouse sequences was considered to avoid designing primers in regions with significant differences across species.

One-step RT-PCR was performed to amplify aliquots of 60-80 ng of RNA using the Qiagen OneStep RT-PCR Kit. The sequences and annealing temperatures of all the primer pairs are provided in Table S1. Finally, the sequence information generated was assembled and analyzed for polymorphisms.

After the mRNA sequence was established, the predicted protein sequence was compared with the human and mouse *RELN* protein sequences using the align option of the web-based UniProt software (http://www.uniprot.org/). The effects on protein sequence and secondary structures of the mutations that had been identified by the sequencing analysis were predicted using the SWISS-MODEL software [21-23].

### Confirmation and genotyping of the causal mutation

After identifying the putative causal mutation in the mRNA sequence, we confirmed its direct association with the studied phenotype by analyzing additional samples at the genomic DNA level. DNA was extracted from blood samples collected from six cases, six controls and six mothers of affected animals.

A pair of primers (RELNovine_ex36up: 5’-TTGCCTTCTCCGGTTTAATG and RELNovine_ex36dn: 5’-AGGGATTTGTGATGCTGGAC) were designed to amplify a 498-bp fragment of the gene sequence containing the 31-bp deletion that was identified as the possible causal mutation. The amplicons were purified by ExoSAP-IT (UBS Corporation) treatment and were dideoxy-sequenced in both directions with the Big Dye Terminator Cycle Sequencing Kit v3.1 (Applied Biosystems) with the same primers used for fragment amplification. The sequence data were analyzed with SeqScape v2.5 software (Applied Biosystems). 

### Functional analysis of the 31-bp deletion: quantification of RELN mRNA by qRT-PCR

To quantify the levels of *RELN* mRNA in affected lambs and to compare them with unaffected animals, we performed two qRT-PCR amplifications in six LCH cases and six controls. The qRT-PCR assay was designed to amplify two different regions of the ovine *RELN* gene: a shared region which included 139 bp of the third reelin repeat, and the mutated region which include the four reelin-specific repeat encompassing the deletion. 

Total RNA was isolated from the brains of six controls and six cases using TRIzol® Reagent and the PureLink™ RNA Mini Kit (Invitrogen) according to the manufacturer's instructions. The high capacity cDNA Reverse Transcription kit (Applied Biosystems) was used to synthesize DNA according to the manufacturer's instructions. Quantitative reverse transcription polymerase chain reaction was performed using the StepOne™ Real-Time PCR System (Applied Biosystems) with Power SYBR® Green PCR Master Mix according to the manufacturer's instructions (see Table S2 for the primer sequences). The *RELN* gene transcript levels were calculated using the relative standard curve method normalized to glyceraldehyde-3-phosphate dehydrogenase (GAPDH).

### Functional analysis of the 31-bp deletion: Western blot analysis

A Western blot was performed to confirm that the 31-bp deletion identified in the affected individuals affected reelin expression. We analyzed the protein in brains and sera from affected and unaffected animals. Samples (0.1 g) of sheep frontal cortex were homogenized (10% w/v) in 50 mM Tris-HCl pH 7.4, 150 mM NaCl, 0.5% Triton X-100 and 0.5% Nonidet P-40 containing a cocktail of protease inhibitors [24]. The homogenates were sonicated and centrifuged at 20,000×g for 20 min at 4°C, and the supernatants collected and frozen at -80°C. Sheep brain extracts (30 μg) and serum samples (0.8 µl) were incubated with 6× SDS-PAGE sample buffer at 98°C for 3 min and then resolved using 6% SDS-polyacrylamide gel electrophoresis (SDS-PAGE). The proteins were blotted onto nitrocellulose membranes and incubated with the monoclonal mouse anti-Reelin 142 antibody (1:500 dilution, Merck Millipore) and with a secondary antibody (1:4000 dilution, horseradish peroxidase (HRP)-conjugated anti-mouse IgG, (Sigma Chemicals). The signals were visualized with the HRP chemiluminescent substrate Luminata^TM^ Forte (Merck Millipore) in a Luminescent Image Analyzer LAS-1000 Plus (Fujifilm), and analyzed using the Science Lab Image Gauge v3.0 software (Fujifilm). 

## Results

### Pedigree analysis

**Figure 1:**
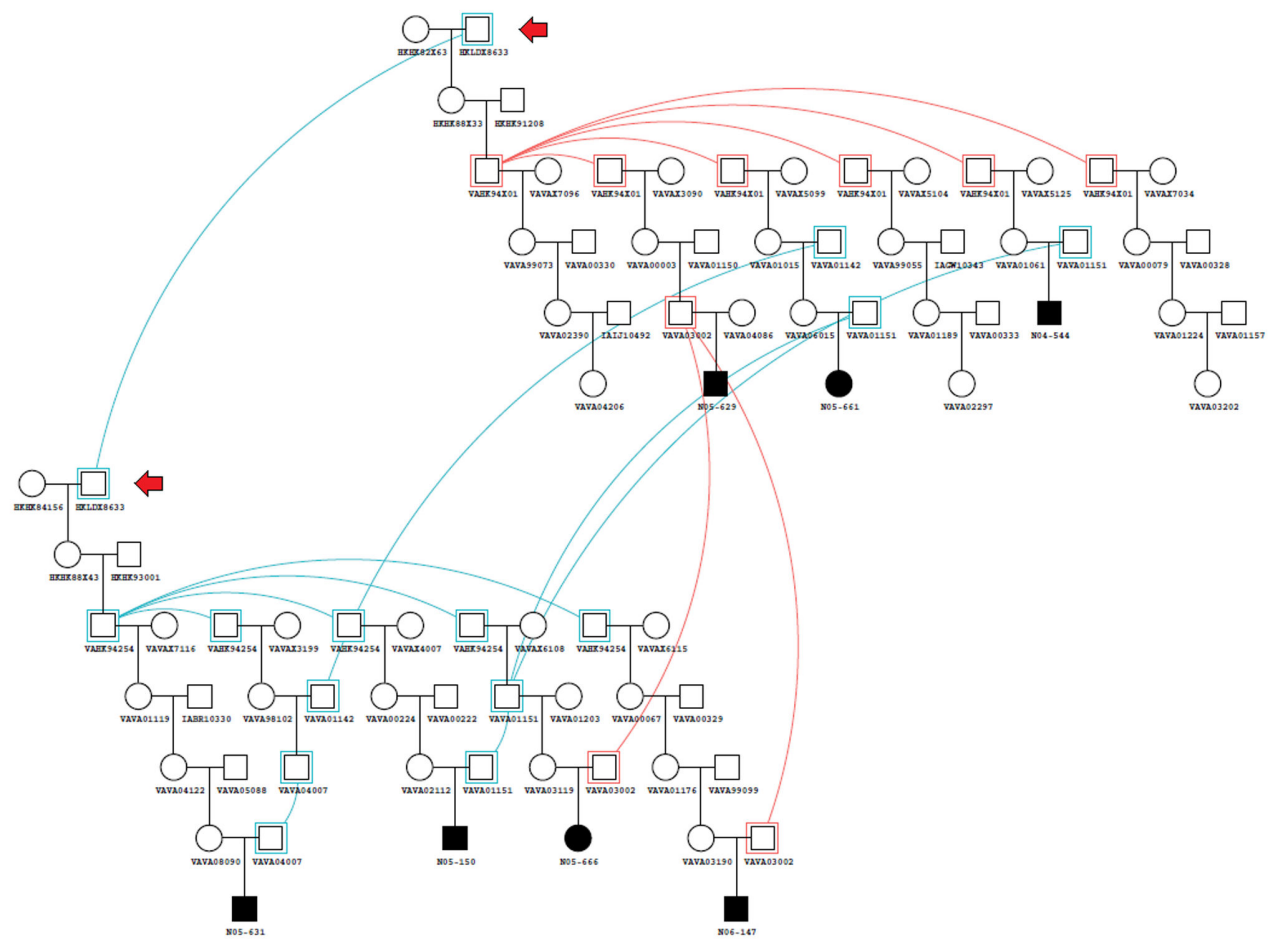
Pedigree displaying LCH in Churra sheep. The arrow indicates the ram identified as the common founder animal for the LCH disease. The lines indicate the inbreeding lops. Cases are indicated as solid symbols.

The pedigree of the affected individuals (Figure 1) was constructed to confirm the mode of inheritance previously described [10] and to search for the common founder of LCH in this flock. The breeding history of the affected families was consistent with a monogenic autosomal recessive pattern of inheritance. The parents and half-siblings of the affected animals did not show clinical signs of the disease. The parents of the affected animals were classified as obligate carriers.

An analysis of the pedigree data revealed that the LCH-affected individuals were descended from a single ram founder (HKLD8633) via either the paternal or maternal line (Figure 1). This male was born in 1986, and the disease was first recognized in 2004, four generations later.

### Genome-wide association and runs of homozygosity studies

**Figure 2:**
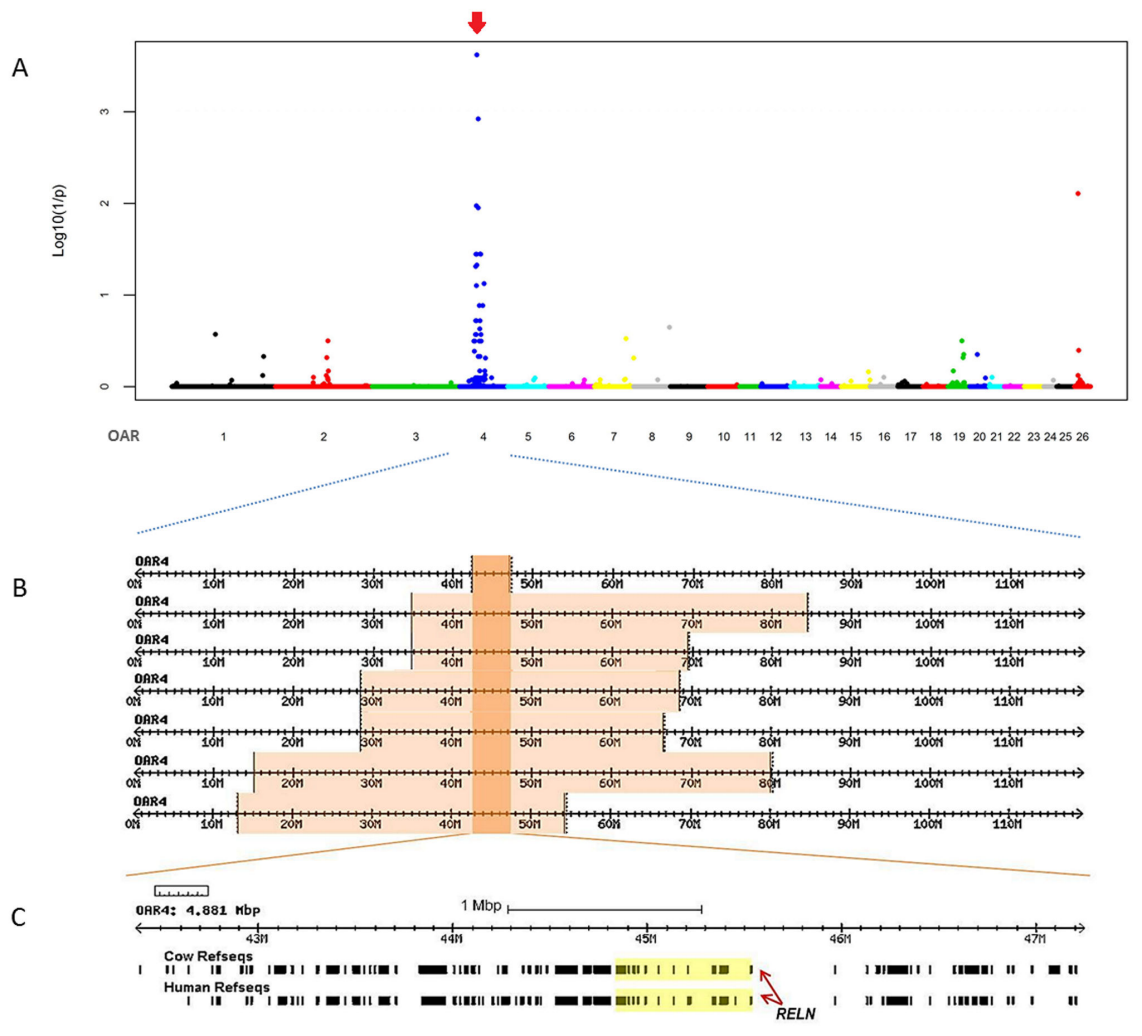
GWA and homozygosity mapping analyses for ovine LCH. (A) Manhattan plot resulting from the case-control association analysis performed with 7 affected animals and 33 controls. The X-axis shows the positions of the genome analyzed across the 26 ovine autosomes, whereas the Y-axis represents the -log_10_ P-values obtained after 100,000 permutations using PLINK. Alternating colors mark the limits between autosomes. The arrow indicates the most significant association identified, which was located on OAR4 (at 42,810,217 bp position). (B) Homozygosity mapping of the LCH mutation. The analysis of SNP genotypes from affected lambs indicated that they all shared an extended overlapping homozygous region on OAR 4 (indicated by orange blocks). The common haplotype block, where the causative mutation is located, expands between 42.369 and 47.251 Mb on OAR4 (indicated by the red box). (C) Gene content of the 4.8-Mb homozygosity block interval shared by the affected individuals based on the comparative genomic map with the orthologous regions in human and cow and including the *RELN* gene.

The GWA study revealed that the strongest association with the LCH phenotype localized to sheep chromosome 4 (OAR4) (Figure 2). The SNP showing the most highly significant association was OAR4_45088426, which is located at position 42,810,217 bp in the OARv3.1 ovine genome sequence. After 100,000 permutations were performed, this SNP showed a genome-wide corrected P-value of 2.4×10^-4^. 

The homozygosity mapping approach that was performed later to narrow the region containing the LCH mutation was chosen on the assumption that the affected lambs were identical by descent (IBD) for the causative mutation and the flanking chromosomal segments. The seven genotyped cases showed a single, large homozygous consensus region on OAR4 that contained 91 SNP markers corresponding to a 4.8-Mb interval from 42.369-47.251 Mb (Figure 2). Analyzing the data from the 10 unrelated healthy Churra controls did not reveal any homozygous shared regions across the genome that were greater than 300 Kb. Because of the limited annotation of the ovine genome assembly (v 3.1), we inferred the gene annotation of the mapped interval from the corresponding bovine and human orthologous intervals. The ovine LCH interval corresponds to segments on bovine chromosome (BTA) 4 and human chromosome (HSA) 7. A careful inspection of the BTA4 and HSA7 genes and database searches for their presumed functions indicated that the gene that encodes the reelin protein was the most promising functional candidate gene. *RELN* is located within the critical identified intervals: at 44.6 Mb on OAR4, at 44.8 Mb on BTA4 and at 103.1 Mb on HSA7. The reelin protein plays critical roles in neuronal migration and layer formation [25] and has been associated with the human LIS forms LIS2 and Norman-Roberts syndrome [4].

### Complete coding sequence of the ovine RELN gene

Because no reference sequence of the ovine *RELN* gene was initially available, the sequencing analysis of the specific mRNA of this gene in a control lamb allowed us to obtain the complete cDNA of the ovine *RELN* gene (GenBank acc. no. KC590614).

The BLAST comparison of the sequenced cDNA and the genomic sequence obtained from the ovine genome assembly v3.1 using the Spidey program showed an overall identity of 99% with 95% of mRNA coverage. The comparison between the cDNA and genomic DNA sequences allowed us to predict the structure of the coding sequence at the genomic level, resulting in 64 exons. 

The sheep *RELN* cDNA sequence encodes a predicted protein of 3460 amino acids. The UniProt software (http://www.uniprot.org/) was used to compare the predicted amino acid sequence from the ovine *RELN* gene with several available sequences, including human (P78509), mouse (Q60841) and rat (P58751) (Figure S1). The ovine *RELN* protein sequence showed 97% homology with the human protein and 95% homology with the mouse and rat sequences, suggesting high conservation of this protein across species. 

### DNA variant scanning and identification of the causal mutation

To identify the causal mutation of the studied disease, the entire *RELN* coding sequence from an affected lamb was sequenced. We amplified 9986 bp, corresponding to 96.21% of the coding region previously amplified in the control sample.

The sequence comparison between the control and affected samples revealed the presence of six SNPs and one 31-bp deletion (Table 1). For the two SNPs that produced amino acid changes, the LCH-affected lamb shared the heterozygous state with the normal lamb (p.Leu15Gln) or was homozygous for the wild-type allele exhibited by 8 normal animals and the reference sequence of the ovine assembly at this position (p.Met2670Ile). In contrast, the identified 31-bp deletion (c.5410_5440del) was present in a homozygous form in the LCH lamb (c.5410_5440del) but not in the control animal. Based on the genomic structure obtained by a comparison of the cDNA and the gDNA, this mutation occurred within the sequence of predicted exon 36 of the ovine *RELN* gene. This mutation was predicted to cause a shift in the open reading frame of the *RELN* coding sequence and create a stop codon at position 1817. This mutation would lead to a truncated protein of 1817 amino acids (1803 amino acids of normal reelin followed by 14 missense amino acids and a premature termination codon) *vs.* the normal 3460-amino-acid-long RELN protein. Due to its highly disruptive effect on the composition and structure of the resulting protein, this 31-bp deletion was identified as possibly directly responsible for the disease under study. 

**Table 1:** DNA variants found by cDNA sequencing of the ovine *RELN* gene in a case and a control.

**Mutation ID**	**Control**	**Case**	Reference*	**Protein level**
c.44ta	t/a	t/a	t	p.Leu15Gln
c.2676ag	a/a	g/g	a	p.=
c.5410_5440del	-	del	-	p. 0
c.5862ct	c/t	c/c	c	p.=
c8010gc	g/c	g/g	g	p.Met2670Ile
c8754tc	t/c	t/t	t	p.=
c.9153ag	a/g	a/a	a	p.=

The effect of each DNA variant on the protein sequence is indicated in the last column.

* Reference based on the nucleotide found for this position in the Ovine Genome Assembly v3.1.

### Confirmation and genotyping of the causal mutation

**Figure 3:**
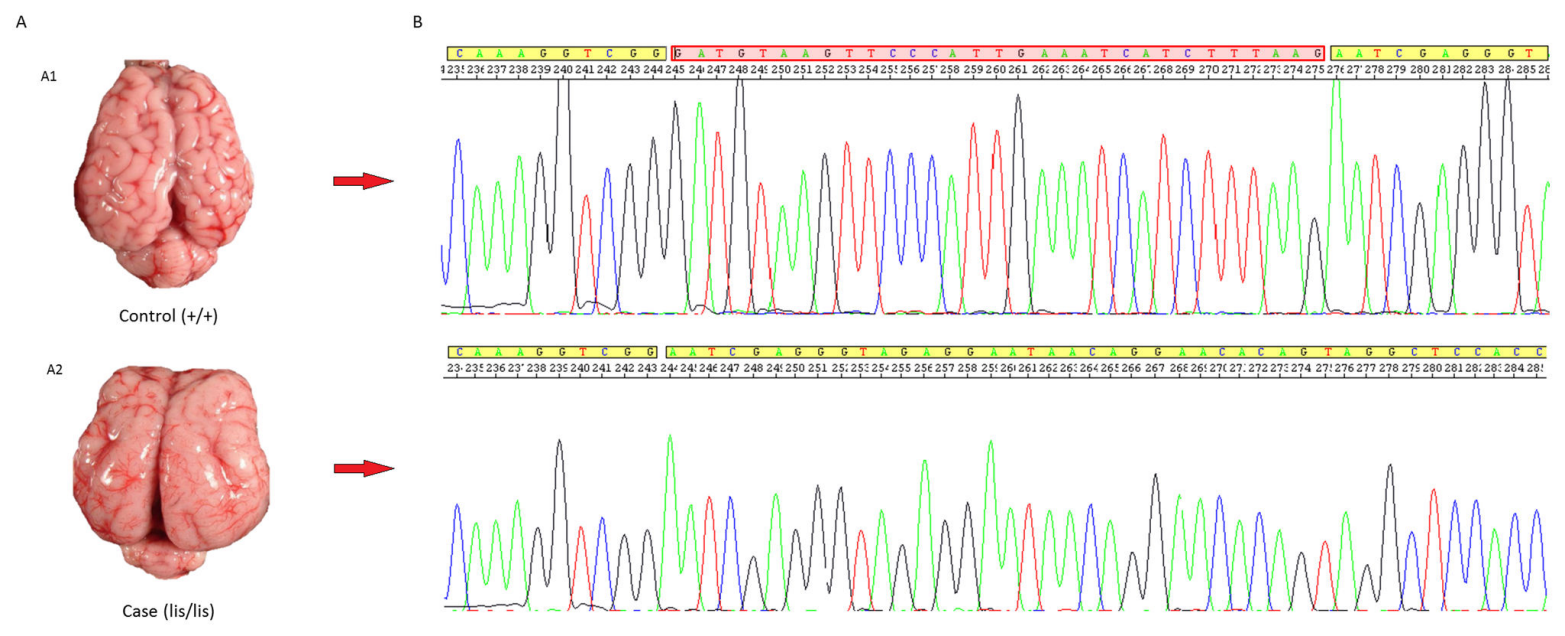
Identification of the causative mutation for LCH in sheep. (A) LCH phenotype in Spanish Churra sheep. (A1) Normal newborn lamb brain. (A2) LCH-affected lamb brain. (B) Electropherograms including the mutated region at exon 36 of *RELN* in the normal and LCH-affected lambs. The bases within the red square are included in the 31-bp deletion (c.5410_5440del) detected in the LCH-affected lambs.

To confirm a direct association between the c.5410_5440del allelic variant and the LCH phenotype, we searched for a correspondence between the genotype of this deletion and the gDNA sequences of affected and carrier animals. Using DNA samples, we performed PCR amplifications of putative exon 36 for six cases, six unrelated controls and six mothers of affected lambs. The resulting amplicon was sequenced and analyzed with the SeqScape v2.5 software (Applied Biosystems). All the samples from the LCH-affected animals were homozygous for the identified 31-bp deletion (c.5410_5440del/c.5410_5440del or Lis/Lis); the mothers were heterozygous for this mutation (Lis/+); and the controls from different flocks were homozygous (+/+) for the wild-type allele (Figure 3). 

### Functional analysis of the 31-bp deletion

**Figure 4:**
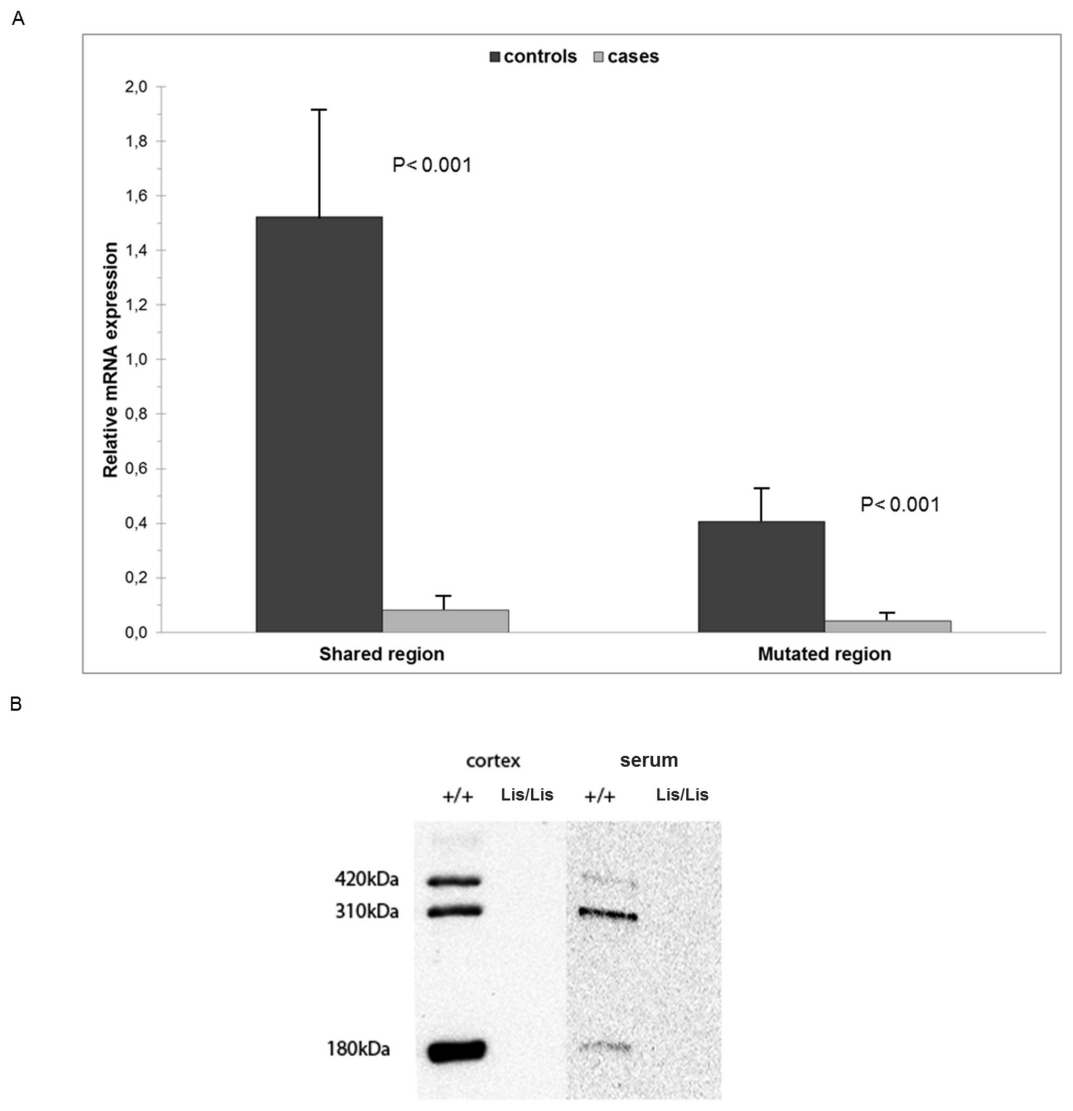
Functional evaluation of the 31-bp deletion (c.5410_5440del) in the ovine *RELN* gene. (A) Expression of *RELN* mRNA analyzed by qRT-PCR. The figure shows the relative expression obtained from two regions of the mRNA: the “Shared region” includes 139 bp of the third reelin repeat, and the “Mutated region” amplifies a fragment that includes the fourth repeat, where the 31-bp deletion was located. The samples were analyzed in triplicate. The values were calculated using relative standard curves and were normalized to GAPDH from the same cDNA preparations and are expressed as the means ± SEM. The specificity of the PCR products was confirmed by melting curve analysis after the qRT-PCR. In both regions significant differences (P < 0.001) in mRNA level were evidenced using the one-tailed Student’s t-test. (B) Western blot immunoprobed for reelin of brain extracts (30 µg) and serum samples (0.8 µl) from control lamb (+/+) and lissencephaly-affected lamb homozygous for the deletion (Lis/Lis).

The levels of *RELN* mRNA in the affected lambs were markedly reduced, with means of 0.08 for the shared region and 0.04 for the mutated region compared with the mean values observed for the control animals (1.52 and 0.40 for the shared and mutated fragments, respectively) (Figure 4A). In both cases, a one-tailed Student’s t-test revealed significant differences in the *RELN* mRNA expression level between the cases and controls (P < 0.001). We also analyzed the protein levels of reelin in the cortex and serum using Western blots (Figure 4B). Three typical reelin-immunoreactive bands of 420 kDa, 310 kDa and 180 kDa with different densities corresponding to the full-length and truncated N-terminal fragments [24] were observed in both the cortex and the serum of healthy lambs. The homozygous Lis/Lis animals showed a lack of reelin in both, tissue and fluids.

## Discussion

We herein demonstrate that LCH in sheep is due to a 31-bp deletion encompassing predicted exon 36 of the ovine *RELN* gene. This mutation was the only abnormality detected by sequencing the entire CDS of the *RELN* gene, which was the best positional and functional candidate in the region highlighted by GWA and homozygosity analyses. In this study, we revealed that homozygous individuals for this mutation lacked the extracellular protein reelin, which has been shown to play a key role in neuronal migration during the embryonic stage and whose absence causes LCH in humans and rodents [4,26]. The discovery of this mutation has allowed screening in the affected flock to identify phenotypically normal individuals carrying the 31-bp deletion to avoid at-risk matings. The implementation of this gene-assisted selection approach has solved the problem of LCH in Churra sheep flocks. 

The results of this work support previous results in different livestock species, in which the use of genomic methodologies has had important practical consequences. High-throughput genotyping platforms allow the rapid control of emerging recessive defects with otherwise major economic and animal welfare implications [11,14]. In this study, 7 LCH cases, 13 related animals and 20 unrelated controls were genotyped using the OvineSNP50 BeadChip. Both the case-control association analysis and the run of homozygosity performed with the SNP-chip derived dataset suggested that the same region on chromosome OAR4 was the most likely to harbor the causal mutation responsible for LCH in Churra sheep. Within the homozygous consensus interval on OAR4 (42.4-47.2 Mb), we identified the *RELN* gene as a good functional and positional candidate for the studied phenotype. Based on the recessive mode of inheritance of the disease, which had been deduced based on a segregation analysis of affected pedigrees, a loss-of-function mutation in the coding sequence of the *RELN* gene was predicted.

Sequencing the cDNA of the *RELN* gene allowed us to obtain the complete coding sequence of this gene in a control sample. For the case sample, 96.21% of the sequence was obtained, revealing a deletion of 31 bp in putative exon 36 of the *RELN* gene (c.5410_5440del), which results in the premature termination of the predicted protein and the loss of the final 1643 amino acids of the protein, including the loss of the highly basic C-terminal region [27,28]. The mutation was confirmed at the genomic level, and three genotypes cosegregate with disease status: homozygous mutants (Lis/Lis) were the affected lambs, heterozygous animals (Lis/+) corresponded to the phenotypically normal carriers (ewes and rams of the affected lambs), and the wild-type genotype (+/+) was identified in all the analyzed control individuals. These genotypes completely agreed with the autosomal recessive mode of Mendelian inheritance previously inferred for LCH in Churra sheep [10].

The low amount of mRNA in the LCH brain samples compared with the control samples (identified through the qRT-PCR analysis) may explain the difficulties experienced in the amplification of some regions of the coding sequence in the affected animals. The decreased levels of *RELN* mRNA may be the result of nonsense-mediated mRNA decay (NMD). NMD is a mechanism whereby mRNAs harboring premature termination codons are selectively degraded. This quality-control machinery prevents the production of truncated proteins with dominant-negative or deleterious gain-of-function activities [29] and is the likely reason for the absence of a protein signal in the Western blots of the LCH-affected lambs. Western blots were resolved with the anti-reelin antibody 142, an antibody raised to the N-terminus of reelin, and thus presumably should detect a hypothetical truncated C-terminal reelin.

Lissencephaly is a neuronal migration disorder characterized by a paucity or absence of cerebral sulcation and gyration accompanied by abnormal architecture of the cerebral cortex [30]. This disease comprises a group of brain developmental disorders with a heterogeneous genetic basis [31-33]. Human LCH, an autosomal recessive form of lissencephaly, has been described in two consanguineous pedigrees (OMIM #257320), and two different variants of the *RELN* gene were discovered as causal mutations in these patients [4]. Mutations that inactivate the *RELN* gene produce the majority of the identified mouse mutants with a phenotype similar to LCH, known as *reeler* [34-36]. However, mutant mice with phenotypes indistinguishable from *reeler* have also been identified by disrupting genes such as *Dab1*, *ApoER2* and *Vldlr*, which are involved in the reelin signaling pathway [2,37].

**Figure 5:**
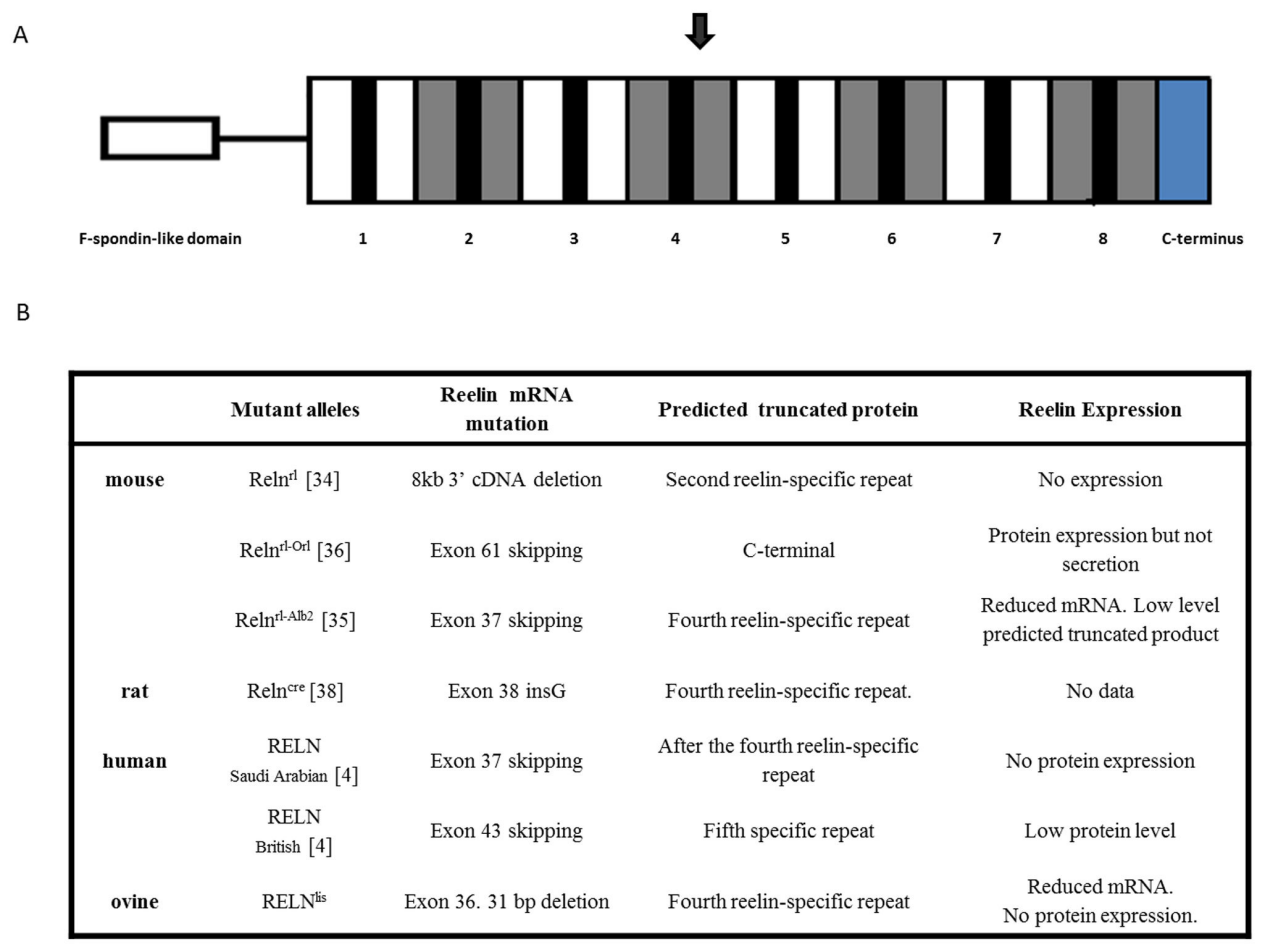
Schematic representation of *RELN* mutant alleles. (A)Predicted wild-type reelin protein consist of a F-spondin-like domain at the amino terminus, followed by 8 reelin repeats, each consisting of an internal EGF-like motif (black) flanked by reelin-specific sequence. The C-terminus includes highly basic aminoacids that appear to be required for secretion. The location of the predicted protein termination due to the ovine deletion is indicated by a black arrow. (B) Table comparing the *RELN* mutant alleles detected in different species indicating the effect of each mutation on reelin activity. The reference for each allele is indicated between square brackets.

Among the known *RELN* mutations, the mouse Albany2 (Reln ^rl-Alb2^) [35], the creeping rat (Reln^cre^) [38] and the human mutation found in a Saudi Arabian [4] pedigree resemble the ovine *RELN* mutation (Figure 5). All these mutations are expected to truncate the fourth specific reelin repeat of the predicted protein. As in the Albany2 *reeler*, the LCH-affected lambs exhibited reduced RNA concentrations, although no experimental protein analysis data was available from this mutant mouse [35]. Western blot analyses of sera from human LCH patients have shown a loss of reelin protein [4] similar to that occurring in the sheep cases reported here. 

The resemblance between the mutations associated with LCH in the four species mentioned above supports the causative nature of the *RELN* gene 31-bp deletion reported here in relation to the LCH phenotype observed in Churra sheep and suggests that this is likely a null allele that is directly related to the LCH disease. 

Development of the human brain requires a complex dynamic process of neuronal migration and positioning. A crucial problem in developmental neurobiology is to understand the genetic and biochemical pathways that regulate production and migration of immature neurons. A detailed understanding of the mechanism of action of genes involved is necessary. Animal models have efficiently been used to understand the mechanisms of action and the pathophysiological abnormalities that result from mutations in genes involved in human lissencephaly [37,38]. A potential long-term goal such the development of target therapies for Human Lissencephaly would not be possible without a detailed understanding of the neuronal migration pathway. 

Reelin protein has been identified as a major determinant of neuronal migration and plays a critical role in neuronal cell maturation during the development of central nervous system. *Reeler* mice mutants have been used as an opportunity to dissect the complex mechanism that underlies the molecular basis of the brain development [34]. Nonetheless, the adult primate brain displays remarkable differences with the rodent brain in the reelin-immunoreactive pattern [39]; therefore accessibility to large animal models maybe of interest in order to a better approach to human disease.

To our knowledge, the LCH sheep is the first large animal model with a confirmed mutation in *RELN*. A large *RELN* mutant model would be a valuable and powerful tool for studies of reelin function in neuronal migration and brain development. Large animal models present many advantages compared with small animal models, such as their greater physiological similarity to human patients and their larger body size [40].

Furthermore, animal models in which reelin expression is altered have provided important information about the actions of this protein in the mature brain. Recent evidence has shown that reelin is involved in modulating synaptic function in adults [41-45]. Several disorders, such as autism, schizophrenia, bipolar disorder, major depression and Alzheimer’s disease, have been associated with abnormal levels of reelin in the serum and brain [46]. In this context, the longer life span and brain physiology of humans show a higher level of resemblance with the ovine model described here than with other small mutant models. Hence, the sheep *RELN* mutant model described in this study would allow a better understanding of reelin synaptic functions and their implications in the neuronal disorders described in the mature brain.

In conclusion, we have identified the causal mutation for ovine LCH in Churra sheep. This mutation (c.5410_5440del) is located in putative exon 36 of the *RELN* gene and leads to the formation of a premature termination codon without protein expression. 

The identification of the causal mutation for LCH in Churra sheep allows us to identify carriers and help breeders avoid at-risk matings between heterozygous animals. Moreover, the identification of *RELN* mutant sheep provides a suitable large model for the study of reelin functions in the developing and mature brain.

## Supporting Information

Figure S1 **Predicted ovine Reelin aminoacid sequence.** Aminoacid sequences of sheep (upper sequence), human (P78509), mouse (Q60841) and rat (P58751) Reelin are aligned using the UniProt software (http://www.uniprot.org/align). The sequence comparison showed a high conservation of this protein across species.
(PDF)

Table S1 **Primers used in the amplification cDNA of the ovine *RELN* gene.** The length of the amplified product and the melting temperature (Tm) of the RT-PCR are also indicated.
(DOCX)

Table S2 **Primers used to quantify the levels of RELN mRNA by qRT-PCR.** The length of the amplified product and the melting temperature (Tm) are also indicated.
(DOCX)
