# Supplementary material for: Identification of a 31-bp Deletion in the RELN Gene Causing Lissencephaly with Cerebellar Hypoplasia in Sheep
Source: PLoS One. 2013 Nov 19;8(11):e81072. doi: 10.1371/journal.pone.0081072 (PMC3834269; doi:10.1371/journal.pone.0081072)
Supplement: Figure S1 — Predicted ovine Reelin aminoacid sequence. Aminoacid sequences of sheep (upper sequence), human (P78509), mouse (Q60841) and rat (P58751) Reelin are aligned using the UniProt software (http://www.uniprot.org/align). The sequence comparison showed a high conservation of this protein across species. (PDF) [file pone.0081072.s001.pdf]

Figure S1. Predicted ovine Reelin aminoacid sequence. Aminoacid sequences of sheep (upper sequence), human (P78509), mouse (Q60841) and rat (P58751) Reelin are aligned using the UniProt software (http://www.uniprot.org/align). The sequence comparison showed a high conservation of this protein across species.

```
CLUSTAL O(1.1.0) multiple sequence alignment


RELN_SHEEP                         MERSSWAPRTFLLAL--LLGATLRARAAVGYYPRFSPFFFLCTHHGELEGDGEQGEVLIS 57
SP|sp|P78509|RELN_HUMAN|RELN_HUMAN MERSGWARQTFLLAL--LLGATLRARAAAGYYPRFSPFFFLCTHHGELEGDGEQGEVLIS 58
SP|sp|Q60841|RELN_MOUSE|RELN_MOUSE MERGCWAPRALVLAVLL-LLATLRARAATGYYPRFSPFFFLCTHHGELEGDGEQGEVLIS 59
SP|sp|P58751|RELN_RAT|RELN_RAT     MERGCWAPRTLVLAVLLLLLATLRARAATGYYPRFSPFFFLCTHHGELEGDGEQGEVLIS 60
                                   ***. ** ::::**:   * ********.*******************************

RELN_SHEEP                         LHIAGHPTYYVPGQEYHVTISTSTFFDGLLVTGLYTSTSVQASQSIGGSNAFGFGIMSDH 117
SP|sp|P78509|RELN_HUMAN|RELN_HUMAN LHIAGNPTYYVPGQEYHVTISTSTFFDGLLVTGLYTSTSVQASQSIGGSSAFGFGIMSDH 118
SP|sp|Q60841|RELN_MOUSE|RELN_MOUSE LHIAGNPTYYVPGQEYHVTISTSTFFDGLLVTGLYTSTSIQSSQSIGGSSAFGFGIMSDH 119
SP|sp|P58751|RELN_RAT|RELN_RAT     LHIAGNPTYYVPGQEYHVTISTSTFFDGLLVTGLYTSTSIQSSQSIGGSSAFGFGIMSDH 120
                                   *****.*********************************:*:*******.**********

RELN_SHEEP                         QFGNQFMCSVVASHVSHLPTTNLSFVWIAPPAGTGCVNFMATATHRGQIIFKDALAQQLC 177
SP|sp|P78509|RELN_HUMAN|RELN_HUMAN QFGNQFMCSVVASHVSHLPTTNLSFIWIAPPAGTGCVNFMATATHRGQVIFKDALAQQLC 178
SP|sp|Q60841|RELN_MOUSE|RELN_MOUSE QFGNQFMCSVVASHVSHLPTTNLSFVWIAPPAGTGCVNFMATATHRGQVIFKDALAQQLC 179
SP|sp|P58751|RELN_RAT|RELN_RAT     QFGNQFMCSVVASHVSHLPTTNLSFVWIAPPAGTGCVNFMATATHRGQVIFKDALAQQLC 180
                                   *************************:**********************:***********

RELN_SHEEP                         EQGAPTEATVHPHLAEIHSNSIILRDDFDSYHQKELNPNIWVECNNCETGEQCGAIMHGN 237
SP|sp|P78509|RELN_HUMAN|RELN_HUMAN EQGAPTDVTVHPHLAEIHSDSIILRDDFDSYHQLQLNPNIWVECNNCETGEQCGAIMHGN 238
SP|sp|Q60841|RELN_MOUSE|RELN_MOUSE EQGAPTEATAYSHLAEIHSDSVILRDDFDSYQQLELNPNIWVECSNCEMGEQCGTIMHGN 239
SP|sp|P58751|RELN_RAT|RELN_RAT     EQGAPTEATAYSHLAEIHSDSVILRDDFDSYHQLELNPNIWAECSNCDTGEQCGTIMHGN 240
                                   ******:.*.: *******:*:*********:* :******.**.**: *****:*****

RELN_SHEEP                         AVTFCEPYGPRELITTGLNTTTASVLQFSIGSGSCRYSYSDPCIIVSYAKNNTADWIQLE 297
SP|sp|P78509|RELN_HUMAN|RELN_HUMAN AVTFCEPYGPRELITTGLNTTTASVLQFSIGSGSCRFSYSDPSIIVLYAKNNSADWIQLE 298
SP|sp|Q60841|RELN_MOUSE|RELN_MOUSE AVTFCEPYGPRELTTTCLNTTTASVLQFSIGSGSCRFSYSDPSITVSYAKNNTADWIQLE 299
SP|sp|P58751|RELN_RAT|RELN_RAT     AVTFCEPYGPRELTTTYLNTTTASVLQFSIGSGSCRFSYSDPSIIVSYAKNNTADWIQLE 300
                                   ************* ** *******************:*****.* * *****:*******

RELN_SHEEP                         KIRAPSNVSTIIHILYLPEDAKGENVQFQWKQENLQVGEVYEACWALDNILIINSAHRQV 357
```

```
SP|sp|P78509|RELN_HUMAN|RELN_HUMAN KIRAPSNVSTIIHILYLPEDAKGENVQFQWKQENLRVGEVYEACWALDNILIINSAHRQV 358
SP|sp|Q60841|RELN_MOUSE|RELN_MOUSE KIRAPSNVSTVIHILYLPEEAKGESVQFQWKQDSLRVGEVYEACWALDNILVINSAHREV 359
SP|sp|P58751|RELN_RAT|RELN_RAT     KIRAPSNVSTIIHILYLPEDAKGENVQFQWKQDSLHVGEVYEACWALDNILVINSAHRQV 360
                                   **********:********:****.*******:.*:***************:******:*

RELN_SHEEP                         VLEDNLDPVDTGNWLFFPGATVKHSCQSDGNSIYFHGNEGSEFNFATTRDVDLSTEDIQE 417
SP|sp|P78509|RELN_HUMAN|RELN_HUMAN VLEDSLDPVDTGNWLFFPGATVKHSCQSDGNSIYFHGNEGSEFNFATTRDVDLSTEDIQE 418
SP|sp|Q60841|RELN_MOUSE|RELN_MOUSE VLEDNLDPVDTGNWLFFPGATVKHSCQSDGNSIYFHGNEGSEFNFATTRDVDLSTEDIQE 419
SP|sp|P58751|RELN_RAT|RELN_RAT     ILEDSLDPVDTGNWLFFPGATVKHSCQSDGNAIYFHGNEGSQLNFATTRDVDLSTEDIQE 420
                                   :***.*************************:*********::******************

RELN_SHEEP                         QWSEEFESQPTGWDIVGAVIGTECGTIESGLSMVFLKDGERKICTPYLDTTGYGNLRFYF 477
SP|sp|P78509|RELN_HUMAN|RELN_HUMAN QWSEEFESQPTGWDVLGAVIGTECGTIESGLSMVFLKDGERKLCTPSMDTTGYGNLRFYF 478
SP|sp|Q60841|RELN_MOUSE|RELN_MOUSE QWSEEFESQPTGWDILGAVVGADCGTVESGLSLVFLKDGERKLCTPYMDTTGYGNLRFYF 479
SP|sp|P58751|RELN_RAT|RELN_RAT     QWSEEFESQPTGWDILGAVVGSECGTIESGLSLVFLKDGERKLCTPYMDTTGYGNLRFYF 480
                                   **************::***:*::***:*****:*********:*** :************

RELN_SHEEP                         VMGGICDPGASHENDVALYAKTEGRKEHITLDTLSYSSYKVPSLVSVVINPDLQTPATKF 537
SP|sp|P78509|RELN_HUMAN|RELN_HUMAN VMGGICDPGNSHENDIILYAKIEGRKEHITLDTLSYSSYKVPSLVSVVINPELQTPATKF 538
SP|sp|Q60841|RELN_MOUSE|RELN_MOUSE VMGGICDPGVSHENDIILYAKIEGRKEHIALDTLTYSSYKVPSLVSVVINPELQTPATKF 539
SP|sp|P58751|RELN_RAT|RELN_RAT     AMGGTCDPGDSHENDVILYAKIEGKKEHIALDTLSYSSYKVPTLVSVVINPELQTPATKF 540
                                   .*** **** *****: **** **:****:****:*******:********:********

RELN_SHEEP                         CLRQKNHQGHNRNVWAIDFFHVLPVLPSTMSHMIQFSINLGCGTQQPGNSISLEFSTNHG 597
SP|sp|P78509|RELN_HUMAN|RELN_HUMAN CLRQKNHQGHNRNVWAVDFFHVLPVLPSTMSHMIQFSINLGCGTHQPGNSVSLEFSTNHG 598
SP|sp|Q60841|RELN_MOUSE|RELN_MOUSE CLRQKSHQGYNRNVWAVDFFHVLPVLPSTMSHMIQFSINLGCGTHQPGNSVSLEFSTNHG 599
SP|sp|P58751|RELN_RAT|RELN_RAT     CLRQKNHQGHNQNVWAVDFFHVLPILPSTMSHMIQFSINLGCGTHQPGNSVSLEFSTNHG 600
                                   *****.***:*:****:*******:*******************:*****:*********

RELN_SHEEP                         RSWSLLHTECLPEICAGPHLPHSTIYSSENYSGWNRITIPLPNAALTRDTRIRWRQTGPI 657
SP|sp|P78509|RELN_HUMAN|RELN_HUMAN RSWSLLHTECLPEICAGPHLPHSTVYSSENYSGWNRITIPLPNAALTRNTRIRWRQTGPI 658
SP|sp|Q60841|RELN_MOUSE|RELN_MOUSE RSWSLLHTECLPEICAGPHLPHSTVYSSENYSGWNRITIPLPNAALTRDTRIRWRQTGPI 659
SP|sp|P58751|RELN_RAT|RELN_RAT     RSWSLLHTECLPEICAGPHLPHSTIYSSENYSGWNRVTIPLPNAALTRDTRIRWRQTGPI 660
                                   ************************:***********:***********:***********

RELN_SHEEP                         LGNMWAIDNVYIGPSCLKFCSGRGQCTRHGCKCDPGFSGPACEMASQTFPMFISESFGSS 717
SP|sp|P78509|RELN_HUMAN|RELN_HUMAN LGNMWAIDNVYIGPSCLKFCSGRGQCTRHGCKCDPGFSGPACEMASQTFPMFISESFGSS 718
SP|sp|Q60841|RELN_MOUSE|RELN_MOUSE LGNMWAIDNVYIGPSCLKFCSGRGQCTRHGCKCDPGFSGPACEMASQTFPMFISESFGSA 719
SP|sp|P58751|RELN_RAT|RELN_RAT     LGNMWAIDNVYIGPSCLKFCSGRGQCTRHGCKCDPGFSGPACEMASQTFPMFISESFGSS 720
                                   ***********************************************************:
```

```
RELN_SHEEP                          RLSSYHNFYSIRGAEVSFGCGVLASGKALVFNKDGRRQLITSFLDSSQSRFLQFTLRLGS 777
SP|sp|P78509|RELN_HUMAN|RELN_HUMAN RLSSYHNFYSIRGAEVSFGCGVLASGKALVFNKDGRRQLITSFLDSSQSRFLQFTLRLGS 778
SP|sp|Q60841|RELN_MOUSE|RELN_MOUSE RLSSYHNFYSIRGAEVSFGCGVLASGKALVFNKDGRRQLITSFLDSSQSRFLQFTLRLGS 779
SP|sp|P58751|RELN_RAT|RELN_RAT     RLSSYHNFYSIRGAEVSFGCGVLASGKALVFNKDGRRQLITSFLDSSQSRFLQFTLRLGS 780
                                   ************************************************************

RELN_SHEEP                         KSVLSTCKAPDQPGEGVLLHYSYDNGITWKLLEHYSYLNYHEPRIISVELPDDARQFGIQ 837
SP|sp|P78509|RELN_HUMAN|RELN_HUMAN KSVLSTCRAPDQPGEGVLLHYSYDNGITWKLLEHYSYLSYHEPRIISVELPGDAKQFGIQ 838
SP|sp|Q60841|RELN_MOUSE|RELN_MOUSE KSVLSTCRAPDQPGEGVLLHYSYDNGITWKLLEHYSYVNYHEPRIISVELPDDARQFGIQ 839
SP|sp|P58751|RELN_RAT|RELN_RAT     KSVLSTCRAPDQPGEGVLLHYSYDNGITWKLLEHYSYLNYHEPRIISVELPDDAKQFGIQ 840
                                   *******:*****************************:.************ **:*****

RELN_SHEEP                         FRWWQPYHSSQGEDVWAIDEIIMTSVLFNSISLDFSNLVEVTQSLGFYLGNVQPYCGHDW 897
SP|sp|P78509|RELN_HUMAN|RELN_HUMAN FRWWQPYHSSQREDVWAIDEIIMTSVLFNSISLDFTNLVEVTQSLGFYLGNVQPYCGHDW 898
SP|sp|Q60841|RELN_MOUSE|RELN_MOUSE FRWWQPYHSSQGEDVWAIDEIVMTSVLFNSISLDFTNLVEVTQSLGFYLGNVQPYCGHDW 899
SP|sp|P58751|RELN_RAT|RELN_RAT     FRWWQPYHSSQGEDVWAIDEILMTSVLFNSISLDFTNLVEVTQSLGFYLGNIQPYCGHDW 900
                                   *********** *********:*************:***************:********

RELN_SHEEP                         TLCFTGDSKLASSMRYVETQSMQIGASYMIQFSLVMGCGQKYTPHMDNQVKLEYSTNHGL 957
SP|sp|P78509|RELN_HUMAN|RELN_HUMAN TLCFTGDSKLASSMRYVETQSMQIGASYMIQFSLVMGCGQKYTPHMDNQVKLEYSTNHGL 958
SP|sp|Q60841|RELN_MOUSE|RELN_MOUSE TLCFTGDSKLASSMRYVETQSMQIGASYMIQFSLVMGCGQKYTPHMDNQVKLEYSANHGL 959
SP|sp|P58751|RELN_RAT|RELN_RAT     TLCFTGDSKLASSMRYVETQSMQIGASYMIQFSLVMGCGQKYTPHMDNQVKLEYSTNHGL 960
                                   *******************************************************:****

RELN_SHEEP                         TWHLVQEECLPSMPSCQEFTSASIYHASEFTQWRRVIVLLPQKTWSSATRFRWSQSYYTA 1017
SP|sp|P78509|RELN_HUMAN|RELN_HUMAN TWHLVQEECLPSMPSCQEFTSASIYHASEFTQWRRVIVLLPQKTWSSATRFRWSQSYYTA 1018
SP|sp|Q60841|RELN_MOUSE|RELN_MOUSE TWHLVQEECLPSMPSCQEFTSASIYHASEFTQWRRVTVVLPQKTWSGATRFRWSQSYYTA 1019
SP|sp|P58751|RELN_RAT|RELN_RAT     TWHLVQDECLPSMPSCQEFTSASIYHASEFTQWRRVTVILPQKTWSGATRFRWSQSYYTA 1020
                                   ******:***************************** *:*******.*************

RELN_SHEEP                         QDEWALDSIYIGQQCPNMCSGHGSCNRGMCRCDQGYQGTECHPEAALPSTIMSDFENPSA 1077
SP|sp|P78509|RELN_HUMAN|RELN_HUMAN QDEWALDSIYIGQQCPNMCSGHGSCDHGICRCDQGYQGTECHPEAALPSTIMSDFENQNG 1078
SP|sp|Q60841|RELN_MOUSE|RELN_MOUSE QDEWALDNIYIGQQCPNMCSGHGSCDHGVCRCDQGYQGTECHPEAALPSTIMSDFENPSS 1079
SP|sp|P58751|RELN_RAT|RELN_RAT     QDEWALDDIYIGQQCPNMCSGHGSCDHGVCRCDQGYQGTECHPEAALPSTIMSDFENPSS 1080
                                   *******.*****************::*:**************************** ..

RELN_SHEEP                         WESDWQEVIGGQIVKPEEGCGVVSSGSSLYFSKAGKRQLVSWDLDTSWVDFVQFYIQIGG 1137
SP|sp|P78509|RELN_HUMAN|RELN_HUMAN WESDWQEVIGGEIVKPEQGCGVISSGSSLYFSKAGKRQLVSWDLDTSWVDFVQFYIQIGG 1138
SP|sp|Q60841|RELN_MOUSE|RELN_MOUSE WESDWQEVIGGEVVKPEQGCGVVSSGSSLYFSKAGKRQLVSWDLDTSWVDFVQFYIQIGG 1139
```

```
SP|sp|P58751|RELN_RAT|RELN_RAT      WDSDWQEVIGGEVVKPEQGCGVVSSGSSLYFSKAGKRQLVSWDLDTSWVDFVQFYIQIGG 1140
                                    *:*********::****:****:*************************************

RELN_SHEEP                          ESSACNKPDSREEGVLLQYSNNGGIQWHLLAEMYFSDFSKPRFVYLELPAAAKTPCTRFR 1197
SP|sp|P78509|RELN_HUMAN|RELN_HUMAN ESASCNKPDSREEGVLLQYSNNGGIQWHLLAEMYFSDFSKPRFVYLELPAAAKTPCTRFR 1198
SP|sp|Q60841|RELN_MOUSE|RELN_MOUSE ESAACNKPDSREEGILLQYSNNGGIQWHLLAEMYFSDFSKPRFVYLELPAAAKTPCTRFR 1199
SP|sp|P58751|RELN_RAT|RELN_RAT      ESAACNKPDSREEGVLLQYSNNGGIQWHLLAEMYFSDFGKPRFVYLELPAAAKTPCTRFR 1200
                                    **::**********:***********************.*********************

RELN_SHEEP                          WWQPVFSGEDYDQWAVDDIIILSEKQKQIIPVVNPTLPQNFYEKPAFDYPMNQMSVWLML 1257
SP|sp|P78509|RELN_HUMAN|RELN_HUMAN WWQPVFSGEDYDQWAVDDIIILSEKQKQIIPVINPTLPQNFYEKPAFDYPMNQMSVWLML 1258
SP|sp|Q60841|RELN_MOUSE|RELN_MOUSE WWQPVFSGEDYDQWAVDDIIILSEKQKQVIPVVNPTLPQNFYEKPAFDYPMNQMSVWLML 1259
SP|sp|P58751|RELN_RAT|RELN_RAT      WWQPVFSGEDYDQWAVDDIIILSEKQKQVIPVVNPTLPQNFYEKPAFDYPINQMSVWLML 1260
                                    ****************************:***:*****************:*********

RELN_SHEEP                          ANEGMVKNETFCSATPSAMVFGKSDGDRFAVTRDLTLKPGYVLQFKLNIGCANQFSSAAP 1317
SP|sp|P78509|RELN_HUMAN|RELN_HUMAN ANEGMVKNETFCAATPSAMIFGKSDGDRFAVTRDLTLKPGYVLQFKLNIGCANQFSSTAP 1318
SP|sp|Q60841|RELN_MOUSE|RELN_MOUSE ANEGMAKNDSFCATTPSAMVFGKSDGDRFAVTRDLTLKPGYVLQFKLNIGCTSQFSSTAP 1319
SP|sp|P58751|RELN_RAT|RELN_RAT      ANEGMAKNDSFCATTPSAMVFGKSDGDRFAVTRDLTLKPGYVLQFKLNIGCASQFSSTAP 1320
                                    *****.**::**::*****:*******************************:.****:**

RELN_SHEEP                          VLLQYSHDAGMSWFLVKEGCYPASAGKGCEGNSRELSEPTMYHTGDFEEWTRITIVIPRS 1377
SP|sp|P78509|RELN_HUMAN|RELN_HUMAN VLLQYSHDAGMSWFLVKEGCYPASAGKGCEGNSRELSEPTMYHTGDFEEWTRITIVIPRS 1378
SP|sp|Q60841|RELN_MOUSE|RELN_MOUSE VLLQYSHDAGMSWFLVKEGCFPASAGKGCEGNSRELSEPTVYYTGDFEEWTRITIAIPRS 1379
SP|sp|P58751|RELN_RAT|RELN_RAT      VLLQYSHDAGMSWFLVKEGCFPASAGKGCEGNSRELSEPTVYYTGDFEEWTRVTIAIPRS 1380
                                    ********************:*******************:*:*********:**.****

RELN_SHEEP                          LASSKTRFRWIQESSSQKNVPPFGLDGVYISEPCPSYCSGHGDCVSGVCFCDLGYTAAQG 1437
SP|sp|P78509|RELN_HUMAN|RELN_HUMAN LASSKTRFRWIQESSSQKNVPPFGLDGVYISEPCPSYCSGHGDCISGVCFCDLGYTAAQG 1438
SP|sp|Q60841|RELN_MOUSE|RELN_MOUSE LASSKTRFRWIQESSSQKNVPPFGLDGVYISEPCPSYCSGHGDCISGVCFCDLGYTAAQG 1439
SP|sp|P58751|RELN_RAT|RELN_RAT      LASSKTRFRWIQESSSQKNVPPFGLDGVYISEPCPSYCSGHGDCISGVCFCDLGYTAAQG 1440
                                    ********************************************:***************

RELN_SHEEP                          TCVSIVPNHSEMFDRFEGKLSPLWYKITGGQVGTGCGTLNDGKSLYFSGPGKREARTVPL 1497
SP|sp|P78509|RELN_HUMAN|RELN_HUMAN TCVSNVPNHNEMFDRFEGKLSPLWYKITGAQVGTGCGTLNDGKSLYFNGPGKREARTVPL 1498
SP|sp|Q60841|RELN_MOUSE|RELN_MOUSE TCVSNTPNHSEMFDRFEGKLSPLWYKITGGQVGTGCGTLNDGRSLYFNGLGKREARTVPL 1499
SP|sp|P58751|RELN_RAT|RELN_RAT      TCVSNIPNHSEMFDRFEGKLSPLWYKISGGQVGTGCGTLSDGRSLYFNGLGKREARTVPL 1500
                                    ****  ***.*****************:*.*********.**:****.* **********

RELN_SHEEP                          DTRNIRLVQFYIQIGSKTSGITCIKPRARNEGLVVQYSNDNGILWHLLRELDFMSFLEPQ 1557
```

```
SP|sp|P78509|RELN_HUMAN|RELN_HUMAN DTRNIRLVQFYIQIGSKTSGITCIKPRTRNEGLIVQYSNDNGILWHLLRELDFMSFLEPQ 1558
SP|sp|Q60841|RELN_MOUSE|RELN_MOUSE DTRNIRLVQFYIQIGSKTSGITCIKPRARNEGLVVQYSNDNGILWHLLRELDFMSFLEPQ 1559
SP|sp|P58751|RELN_RAT|RELN_RAT     DTRNIRLVQFYIQIGSKTSGITCIKPRARNEGLVVQYSNDNGILWHLLRELDFLSFLEPQ 1560
                                   ***************************:*****:*******************:******

RELN_SHEEP                         IISIDLPREAKTPATAFRWWQPQHGKHSAQWALDDVLIGMNDSSQTGFQDKFDGSLDLQA 1617
SP|sp|P78509|RELN_HUMAN|RELN_HUMAN IISIDLPQDAKTPATAFRWWQPQHGKHSAQWALDDVLIGMNDSSQTGFQDKFDGSIDLQA 1618
SP|sp|Q60841|RELN_MOUSE|RELN_MOUSE IISIDLPREAKTPATAFRWWQPQHGKHSAQWALDDVLIGVNDSSQTGFQDKFDGSIDLQA 1619
SP|sp|P58751|RELN_RAT|RELN_RAT     IISIDLPREAKTPATAFRWWQPQHGKHSAQWALDDVLIGVNDSSQTGFQDKFDGSIDLQA 1620
                                   *******::******************************:***************:****

RELN_SHEEP                         NWYRIQGGQVDIDCLSMDTALIFAENIGKPRYAETWDFHVSASTFLQFEMSMGCSKPFSD 1677
SP|sp|P78509|RELN_HUMAN|RELN_HUMAN NWYRIQGGQVDIDCLSMDTALIFTENIGKPRYAETWDFHVSASTFLQFEMSMGCSKPFSN 1678
SP|sp|Q60841|RELN_MOUSE|RELN_MOUSE NWYRIQGGQVDIDCLSMDTALIFTENIGKPRYAETWDFHVSASSFLQFEMNMGCSKPFSG 1679
SP|sp|P58751|RELN_RAT|RELN_RAT     NWYRIQGGQVDIDCLSMDTALIFTENIGKPRYAETWDFHVSASSFLQFDMSMGCSKPFSA 1680
                                   ***********************:*******************:****:*.********

RELN_SHEEP                         SHSVQLQYSLNNGRDWHLVTEECVPPTIGCLHYTESSIYTSERFQNWKRITVYLPLSTIS 1737
SP|sp|P78509|RELN_HUMAN|RELN_HUMAN SHSVQLQYSLNNGKDWHLVTEECVPPTIGCLHYTESSIYTSERFQNWKRITVYLPLSTIS 1738
SP|sp|Q60841|RELN_MOUSE|RELN_MOUSE AHGIQLQYSLNNGKDWQLVTEECVPPTIGCVHYTESSTYTSERFQNWRRVTVYLPLATNS 1739
SP|sp|P58751|RELN_RAT|RELN_RAT     THSVQLQYSLNNGKDWHPVTEECVPPTIGCVHYTESSTYTSERFQNWRRVTVYLPLATNS 1740
                                   :*.:*********:**: ************:****** *********:*:******:* *

RELN_SHEEP                         PRTRFRWIQANYTVGADSWAIDNVVLASGCPWMCSGRGICDAGRCVCDRGFGGAYCVPVI 1797
SP|sp|P78509|RELN_HUMAN|RELN_HUMAN PRTRFRWIQANYTVGADSWAIDNVVLASGCPWMCSGRGICDAGRCVCDRGFGGPYCVPVV 1798
SP|sp|Q60841|RELN_MOUSE|RELN_MOUSE PRTRFRWIQTNYTVGADSWAIDNVILASGCPWMCSGRGICDSGRCVCDRGFGGPFCVPVV 1799
SP|sp|P58751|RELN_RAT|RELN_RAT     PRTRFRWIQANYTMGADAWAIDNVLLASGCPWLCSGRGICDSGRCVCDRGFGGPFCVPVV 1800
                                   *********:***:***:******:*******:********:*********** :****:

RELN_SHEEP                         PLPSILKDDFNGNLHPDLWPEVYGAERGNLNGETIKSGTSLIFKGEGLRMLISRDLDCTN 1857
SP|sp|P78509|RELN_HUMAN|RELN_HUMAN PLPSILKDDFNGNLHPDLWPEVYGAERGNLNGETIKSGTSLIFKGEGLRMLISRDLDCTN 1858
SP|sp|Q60841|RELN_MOUSE|RELN_MOUSE PLPSILKDDFNGNLHPDLWPEVYGAERGNLNGETIKSGTCLIFKGEGLRMLISRDLDCTN 1859
SP|sp|P58751|RELN_RAT|RELN_RAT     PLPSILKDDFNGNLHPDLWPEVYGAERGNLNGETIKSGTSLIFKGEGLRMLISRDLDCTN 1860
                                   ***************************************.********************

RELN_SHEEP                         TMYVQFSLRFIAKSTPERSHSILLQFSINGGITWHLMDEFYFPQTTNILFINVPLPYTAQ 1917
SP|sp|P78509|RELN_HUMAN|RELN_HUMAN TMYVQFSLRFIAKSTPERSHSILLQFSISGGITWHLMDEFYFPQTTNILFINVPLPYTAQ 1918
SP|sp|Q60841|RELN_MOUSE|RELN_MOUSE TMYVQFSLRFIAKGTPERSHSILLQFSVSGGVTWHLMDEFYFPQTTSILFINVPLPYGAQ 1919
SP|sp|P58751|RELN_RAT|RELN_RAT     TMYVQFSLRFIAKGTPERSHSILLQSSINGGVTWRLMDEFYFPQTTSILFINVPLPYSAQ 1920
                                   *************.*********** *:.**:**:***********.********** **
```

```
RELN_SHEEP                         TNATRFRLWQPYNNGKKEEIWIVDDFIIDGNNLNNPVMLLDTFDFGPREDNWFFYPGGNI 1977
SP|sp|P78509|RELN_HUMAN|RELN_HUMAN TNATRFRLWQPYNNGKKEEIWIVDDFIIDGNNVNNPVMLLDTFDFGPREDNWFFYPGGNI 1978
SP|sp|Q60841|RELN_MOUSE|RELN_MOUSE TNATRFRLWQPYNNGKKEEIWIIDDFIIDGNNLNNPVLLLDTFDFGPREDNWFFYPGGNI 1979
SP|sp|P58751|RELN_RAT|RELN_RAT     TNATRFRLWQPYNNGKKEEIWIIDDFIIDGDNLNNPVMLLDTFDFGPREDNWFFYPGGNI 1980
                                   **********************:*******:*:****:**********************

RELN_SHEEP                         GLYCPYSSKGAPEEDSAMVFVSNEVGEHSITTRDLNVNENTIIQFEINVGCSTDSSSADP 2037
SP|sp|P78509|RELN_HUMAN|RELN_HUMAN GLYCPYSSKGAPEEDSAMVFVSNEVGEHSITTRDLNVNENTIIQFEINVGCSTDSSSADP 2038
SP|sp|Q60841|RELN_MOUSE|RELN_MOUSE GLYCPYSSKGAPEEDSAMVFVSNEVGEHSITTRDLSVNENTIIQFEINVGCSTDSSSADP 2039
SP|sp|P58751|RELN_RAT|RELN_RAT     GLYCPYSSKGAPEEDSAMVFVSNEIGEHSITTRDLSVNENTIIQFEINVGCSTDSSSADP 2040
                                   ************************:**********.************************

RELN_SHEEP                         VRLEFSRDFGATWHLLLPLCYHSSGHVSSLCSTEHHPSSTYYAGTTQGWRREVVHFGKLH 2097
SP|sp|P78509|RELN_HUMAN|RELN_HUMAN VRLEFSRDFGATWHLLLPLCYHSSSHVSSLCSTEHHPSSTYYAGTMQGWRREVVHFGKLH 2098
SP|sp|Q60841|RELN_MOUSE|RELN_MOUSE VRLEFSRDFGATWHLLLPLCYHSSSLVSSLCSTEHHPSSTYYAGTTQGWRREVVHFGKLH 2099
SP|sp|P58751|RELN_RAT|RELN_RAT     VRLEFSRDFGATWHLLLPLCYHSSSLVSSLCSTEHHPSSTYYAGTTQGWRREVVHFGKLH 2100
                                   ************************. ******************* **************

RELN_SHEEP                         LCGSVRFRWYQGFYSAGSQPVTWAIDNVYIGPQCEEMCNGHGSCINGTKCICDPGYSGPT 2157
SP|sp|P78509|RELN_HUMAN|RELN_HUMAN LCGSVRFRWYQGFYPAGSQPVTWAIDNVYIGPQCEEMCNGQGSCINGTKCICDPGYSGPT 2158
SP|sp|Q60841|RELN_MOUSE|RELN_MOUSE LCGSVRFRWYQGFYPAGSQPVTWAIDNVYIGPQCEEMCYGHGSCINGTKCICDPGYSGPT 2159
SP|sp|P58751|RELN_RAT|RELN_RAT     LCGSVRFRWYQGFYPAGSQPVTWAIDNVYIGPQCEEMCCGHGSCVNGTKCICDPGYSGPT 2160
                                   ************** *********************** *:***:***************

RELN_SHEEP                         CKISTKNPDFLKDDFEGQLESDRFLLMSGGKPSRKCGILSSGNNLFFNEDGLRMLMTRDL 2217
SP|sp|P78509|RELN_HUMAN|RELN_HUMAN CKISTKNPDFLKDDFEGQLESDRFLLMSGGKPSRKCGILSSGNNLFFNEDGLRMLMTRDL 2218
SP|sp|Q60841|RELN_MOUSE|RELN_MOUSE CKISTKNPDFLKDDFEGQLESDRFLLMSGGKPSRKCGILSSGNNLFFNEDGLRMLVTRDL 2219
SP|sp|P58751|RELN_RAT|RELN_RAT     CKISTKNPDFLKDDFEGQLESDRFLLMSGGKPSRKCGILSSGNNLFFNEDGLRMLVTRDL 2220
                                   *******************************************************:****

RELN_SHEEP                         DLSHARFVQFFMRLGCGKGVPDPRSQPVLLQYSLNGGLSWSLLQEFLFSNSSNVGRYIAL 2277
SP|sp|P78509|RELN_HUMAN|RELN_HUMAN DLSHARFVQFFMRLGCGKGVPDPRSQPVLLQYSLNGGLSWSLLQEFLFSNSSNVGRYIAL 2278
SP|sp|Q60841|RELN_MOUSE|RELN_MOUSE DLSHARFVQFFMRLGCGKGVPDPRSQPVLLQYSLNGGLSWSLLQEFLFSNSSNVGRYIAL 2279
SP|sp|P58751|RELN_RAT|RELN_RAT     DLSHARFVQFFMRLGCGKGVPDPRSQPVLLQYSLNGGLSWSLLQEFLFSNSSNVGRYIAL 2280
                                   ************************************************************

RELN_SHEEP                         EIPLKARSASTRLRWWQPSENGHFYSPWVIDQILIGGNISGNTVLEDDFTTLDSRKWLLH 2337
SP|sp|P78509|RELN_HUMAN|RELN_HUMAN EIPLKARSGSTRLRWWQPSENGHFYSPWVIDQILIGGNISGNTVLEDDFTTLDSRKWLLH 2338
SP|sp|Q60841|RELN_MOUSE|RELN_MOUSE EMPLKARSGSTRLRWWQPSENGHFYSPWVIDQILIGGNISGNTVLEDDFSTLDSRKWLLH 2339
```

```
SP|sp|P58751|RELN_RAT|RELN_RAT      EMPLKARSGSTRLRWWQPSENGHFYSPWVIDQILIGGNISGNTVLEDDFSTLDSRKWLLH 2340
                                    *:******.****************************************:**********

RELN_SHEEP                          PGGTKMPVCGSTGDALVFIEKASTRYVVTTDIAVNEDSFLQIDFAASCSVTDSCYAIELE 2397
SP|sp|P78509|RELN_HUMAN|RELN_HUMAN  PGGTKMPVCGSTGDALVFIEKASTRYVVSTDVAVNEDSFLQIDFAASCSVTDSCYAIELE 2398
SP|sp|Q60841|RELN_MOUSE|RELN_MOUSE  PGGTKMPVCGSTGDALVFIEKASTRYVVTTDIAVNEDSFLQIDFAASCSVTDSCYAIELE 2399
SP|sp|P58751|RELN_RAT|RELN_RAT      PGGTKMPVCGSTGDALVFIEKASTRYVVTTDIAVNEDSFLQIDFAASCSVTDSCYAIELE 2400
                                    ****************************:**:****************************

RELN_SHEEP                          YSIDLGLSWHPLIRDCLPTNVECSRYHLQRILVSDTFNKWTRITVPLPPYTRSQATRFRW 2457
SP|sp|P78509|RELN_HUMAN|RELN_HUMAN  YSVDLGLSWHPLVRDCLPTNVECSRYHLQRILVSDTFNKWTRITLPLPPYTRSQATRFRW 2458
SP|sp|Q60841|RELN_MOUSE|RELN_MOUSE  YSVDLGLSWHPLVRDCLPTNVECSRYHLQRILVSDTFNKWTRITLPLPSYTRSQATRFRW 2459
SP|sp|P58751|RELN_RAT|RELN_RAT      YSVDLGLSWHPLVRDCLPTNVECSRYHLQRILVSDTFNKWTRITLPLPAYTRSQATRFRW 2460
                                    **:*********:*******************************:*** ***********

RELN_SHEEP                          HQPAPFDKQQTWAIDNVYIGDGCIDMCSGHGRCIQGNCVCDEQWGGLYCDEPETSLPTQL 2517
SP|sp|P78509|RELN_HUMAN|RELN_HUMAN  HQPAPFDKQQTWAIDNVYIGDGCIDMCSGHGRCIQGNCVCDEQWGGLYCDDPETSLPTQL 2518
SP|sp|Q60841|RELN_MOUSE|RELN_MOUSE  HQPAPFDKQQTWAIDNVYIGDGCLDMCSGHGRCVQGSCVCDEQWGGLYCDEPETSLPTQL 2519
SP|sp|P58751|RELN_RAT|RELN_RAT      HQPAPFDKQQTWAIDNVYIGDGCLDMCSGHGRCIQGSCVCDEQWGGLYCDEPETSLPTQL 2520
                                    ***********************:*********:**.*************:*********

RELN_SHEEP                          KDNFNRAPSNQNWLTVNGGKXSTVCGAVASGMALHFSGGCSRLLVTVDLNLTNAEFIQFY 2577
SP|sp|P78509|RELN_HUMAN|RELN_HUMAN  KDNFNRAPSSQNWLTVNGGKLSTVCGAVASGMALHFSGGCSRLLVTVDLNLTNAEFIQFY 2578
SP|sp|Q60841|RELN_MOUSE|RELN_MOUSE  KDNFNRAPSNQNWLTVSGGKLSTVCGAVASGLALHFSGGCSRLLVTVDLNLTNAEFIQFY 2579
SP|sp|P58751|RELN_RAT|RELN_RAT      KDNFNRAPSNQNWLTVNGGKLSTVCGAVASGLALHFSGGCSRLLVTVDLNLTNAEFIQFY 2580
                                    *********.******.*** **********:****************************

RELN_SHEEP                          FMYGCLITPNNRNQGVLLEYSVNGGITWNLLMEIFYDQYSKPGFVNILLPPDAKEIATRF 2637
SP|sp|P78509|RELN_HUMAN|RELN_HUMAN  FMYGCLITPNNRNQGVLLEYSVNGGITWNLLMEIFYDQYSKPGFVNILLPPDAKEIATRF 2638
SP|sp|Q60841|RELN_MOUSE|RELN_MOUSE  FMYGCLITPSNRNQGVLLEYSVNGGITWNLLMEIFYDQYSKPGFVNILLPPDAKEIATRF 2639
SP|sp|P58751|RELN_RAT|RELN_RAT      FMYGCLITPSNRNQGVLLEYSVNGGITWTLLMEIFYDQYSKPGFVNILLPPDAKEIGTRF 2640
                                    *********.******************.***************************.***

RELN_SHEEP                          RWWQPRHDGLDQNDWAIDNVLISGSADQRTVMLDTFSSAPVPQHERSPADAGPVGRIAFD 2697
SP|sp|P78509|RELN_HUMAN|RELN_HUMAN  RWWQPRHDGLDQNDWAIDNVLISGSADQRTVMLDTFSSAPVPQHERSPADAGPVGRIAFD 2698
SP|sp|Q60841|RELN_MOUSE|RELN_MOUSE  RWWQPRHDGLDQNDWAIDNVLISGSADQRTVMLDTFSSAPVPQHERSPADAGPVGRIAFE 2699
SP|sp|P58751|RELN_RAT|RELN_RAT      RWWQPRHDGLDQNDWAIDNVLISGSADQRTVMLDTFSSAPVPQHERSPADAGPVGRIAFD 2700
                                    ***********************************************************:

RELN_SHEEP                          MFMEDKTAVNEHWLFHDDCTVERFCDSPDGVMICGSHDGREVYAVTHDLTPTEGWIMQFK 2757
```

```
SP|sp|P78509|RELN_HUMAN|RELN_HUMAN MFMEDKTSVNEHWLFHDDCTVERFCDSPDGVMLCGSHDGREVYAVTHDLTPTEGWIMQFK 2758
SP|sp|Q60841|RELN_MOUSE|RELN_MOUSE MFLEDKTSVNENWLFHDDCTVERFCDSPDGVMLCGSHDGREVYAVTHDLTPTENWIMQFK 2759
SP|sp|P58751|RELN_RAT|RELN_RAT     MFMEDKTSVNENWVFHDDCTVERFCDSPDGVMLCGSHDGREVYAVTHDLTPTENWIMQFK 2760
                                   **:****:***.*:******************:******************** ******

RELN_SHEEP                         ISVGCKVSEKVTQNQIHVQYSTDFGVSWNYLVPQCLPADAKCSGSVSQPSVFFPTKGWKR 2817
SP|sp|P78509|RELN_HUMAN|RELN_HUMAN ISVGCKVSEKIAQNQIHVQYSTDFGVSWNYLVPQCLPADPKCSGSVSQPSVFFPTKGWKR 2818
SP|sp|Q60841|RELN_MOUSE|RELN_MOUSE ISVGCKVPEKIAQNQIHVQFSTDFGVSWSYLVPQCLPADPKCSGSVSQPSVFFPTEGWKR 2819
SP|sp|P58751|RELN_RAT|RELN_RAT     ISVGCKVPEKIAQNQIHVQFSTDFGVSWSYLVPQCLPADPKCSGTVSQPSVFFPTKGWKR 2820
                                   ******* **::*******:********.********** ****:**********:****

RELN_SHEEP                         ITYPLPESLVGNPVRFRFYQKHSDMQWAIDNFYLGPECLDNCRGHGDCLKEQCICDPGYS 2877
SP|sp|P78509|RELN_HUMAN|RELN_HUMAN ITYPLPESLVGNPVRFRFYQKYSDMQWAIDNFYLGPGCLDNCRGHGDCLREQCICDPGYS 2878
SP|sp|Q60841|RELN_MOUSE|RELN_MOUSE ITYPLPESLTGNPVRFRFYQKYSDVQWAIDNFYLGPGCLDNCGGHGDCLKEQCICDPGYS 2879
SP|sp|P58751|RELN_RAT|RELN_RAT     ITYPLPESLMGNPVRFRFYQKYSDVQWAIDNFYLGPGCLDNCGGHGDCLKEQCICDPGYS 2880
                                   ********* ***********:**:*********** ***** ******:**********

RELN_SHEEP                         GPNCYLTHTLKTFLKERFDSEEIKPDLWMSLEGGSTCTECGILAEDTALYFGGSTVRQAI 2937
SP|sp|P78509|RELN_HUMAN|RELN_HUMAN GPNCYLTHTLKTFLKERFDSEEIKPDLWMSLEGGSTCTECGILAEDTALYFGGSTVRQAV 2938
SP|sp|Q60841|RELN_MOUSE|RELN_MOUSE GPNCYLTHSLKTFLKERFDSEEIKPDLWMSLEGGSTCTECGVLAENTALYFGGSTVRQAI 2939
SP|sp|P58751|RELN_RAT|RELN_RAT     GPHCYLTHTLKTFLKERFDSEEIKPDLWMSLEGGSTCTECGILAENTALYFGGSTVRQAI 2940
                                   **.*****:********************************:***:*************:

RELN_SHEEP                         TQDLDLRGAKFLQYWGRIGSENNMTSCHRPICRKEGVLLDYSTDGGITWTLLHEMDYQKY 2997
SP|sp|P78509|RELN_HUMAN|RELN_HUMAN TQDLDLRGAKFLQYWGRIGSENNMTSCHRPICRKEGVLLDYSTDGGITWTLLHEMDYQKY 2998
SP|sp|Q60841|RELN_MOUSE|RELN_MOUSE TQDLDLRGAKFLQYWGRIGSENNMTSCHRPVCRKEGVLLDFSTDGGITWTLLHEMDFQKY 2999
SP|sp|P58751|RELN_RAT|RELN_RAT     TQDLDLRGAKFLQYWGRIGSENNMTSCHRPVCRKEGVLLDYSKDGGITWTLLHEMDFQKY 3000
                                   ******************************:*********:*.*************:***

RELN_SHEEP                         ISVRHDYILLPEEALTNTTRLRWWQPFVISNGLVVSGVERAQWALDNILIGGAEINPSQL 3057
SP|sp|P78509|RELN_HUMAN|RELN_HUMAN ISVRHDYILLPEDALTNTTRLRWWQPFVISNGIVVSGVERAQWALDNILIGGAEINPSQL 3058
SP|sp|Q60841|RELN_MOUSE|RELN_MOUSE ISVRHDYILLPEGALTNTTRLRWWQPFVISNGLVVSGVERAQWALDNILIGGAEINPSQL 3059
SP|sp|P58751|RELN_RAT|RELN_RAT     ISVRHDYILLPEGALTNTTRLRWWQPFVISNGLVVSGVERAQWALDNILIGGAEINPSQL 3060
                                   ************ *******************:***************************

RELN_SHEEP                         VDTFDDEGTSHEENWSFYPNAVRTAGFCGNPSFHLYWPNKKKDKTHNALSSRELIIQPGY 3117
SP|sp|P78509|RELN_HUMAN|RELN_HUMAN VDTFDDEGTSHEENWSFYPNAVRTAGFCGNPSFHLYWPNKKKDKTHNALSSRELIIQPGY 3118
SP|sp|Q60841|RELN_MOUSE|RELN_MOUSE VDTFDDEGSSHEENWSFYPNAVRTAGFCGNPSFHLYWPNKKKDKTHNALSSRELIIQPGY 3119
SP|sp|P58751|RELN_RAT|RELN_RAT     VDTFDDEGSSHEENWSFYPNAVRTAGFCGNPSFHLYWPNKKKDKTHNALSSRELIIQPGY 3120
                                   ********:***************************************************
```

```
RELN_SHEEP                         MMQFKIVVGCEATSCGDLHSVMLEYTKDARSDSWQLVQSQCLPSSSNSIGCSPFQFHEAT 3177
SP|sp|P78509|RELN_HUMAN|RELN_HUMAN MMQFKIVVGCEATSCGDLHSVMLEYTKDARSDSWQLVQTQCLPSSSNSIGCSPFQFHEAT 3178
SP|sp|Q60841|RELN_MOUSE|RELN_MOUSE MMQFKIVVGCEATSCGDLHSVMLEYTKDARSDSWQLVQTQCLPSSSNSIGCSPFQFHEAT 3179
SP|sp|P58751|RELN_RAT|RELN_RAT     MMQFKIVVGCEATSCGDLHSVMLEYTKDARSDSWQLVQTQCLPSSSNSIGCSPFQFHEAT 3180
                                   ************************************:***********************

RELN_SHEEP                         IYNAVNSSSWKRITIQLPDHVSSSATQFRWIQKGEETEKQSWAIDHVYIGEACPKLCSGH 3237
SP|sp|P78509|RELN_HUMAN|RELN_HUMAN IYNSVNSSSWKRITIQLPDHVSSSATQFRWIQKGEETEKQSWAIDHVYIGEACPKLCSGH 3238
SP|sp|Q60841|RELN_MOUSE|RELN_MOUSE IYNAVNSSSWKRITIQLPDHVSSSATQFRWIQKGEETEKQSWAIDHVYIGEACPKLCSGH 3239
SP|sp|P58751|RELN_RAT|RELN_RAT     IYNAVNSSSWKRITIQLPDHVSSSATQFRWIQKGEETEKQSWAIDHVYIGEACPRLCSGH 3240
                                   ***:**************************************************:*****

RELN_SHEEP                         GYCTTGAVCICDESFQGDDCSIFSHDLPSYIKDNFESARVTEANWETIQGGVIGSGCGQL 3297
SP|sp|P78509|RELN_HUMAN|RELN_HUMAN GYCTTGAICICDESFQGDDCSVFSHDLPSYIKDNFESARVTEANWETIQGGVIGSGCGQL 3298
SP|sp|Q60841|RELN_MOUSE|RELN_MOUSE GYCTTGAVCICDESFQGDDCSVFSHELPSYIKDNFESARVTEANWETIQGGVIGSGCGQL 3299
SP|sp|P58751|RELN_RAT|RELN_RAT     GYCTTGAVCICDESFQGDDCSVFSHELPSYIKDNFESARVTEANWETIQGGAIGSGCGQL 3300
                                   *******:*************:***:*************************.********

RELN_SHEEP                         APYAHGDSLYFNGCQIRQAATKPLDLTRASKIMFVLQIGSTSQTDSCNSDLSGPHTVDKA 3357
SP|sp|P78509|RELN_HUMAN|RELN_HUMAN APYAHGDSLYFNGCQIRQAATKPLDLTRASKIMFVLQIGSMSQTDSCNSDLSGPHAVDKA 3358
SP|sp|Q60841|RELN_MOUSE|RELN_MOUSE APYAHGDSLYFNGCQIRQAATKPLDLTRASKIMFVLQIGSPAQTDSCNSDLSGPHTVDKA 3359
SP|sp|P58751|RELN_RAT|RELN_RAT     APYAHGDSLYFNGCQIRQAATKPLDLTRASKIMFVLQIGSTAQTDSCNSDLSGPHTVDKA 3360
                                   **************************************** :*************:****

RELN_SHEEP                         VLLQYSVNNGITWHVIAQHQPKDFTQAQRVSYNVPLEARMKGVLLRWWQPRHNGTGHDQW 3417
SP|sp|P78509|RELN_HUMAN|RELN_HUMAN VLLQYSVNNGITWHVIAQHQPKDFTQAQRVSYNVPLEARMKGVLLRWWQPRHNGTGHDQW 3418
SP|sp|Q60841|RELN_MOUSE|RELN_MOUSE VLLQYSVNNGITWHVIAQHQPKDFTQAQRVSYNVPLEARMKGVLLRWWQPRHNGTGHDQW 3419
SP|sp|P58751|RELN_RAT|RELN_RAT     VLLQYSVNNGITWHVIAQHQPKDFTQAQRVSYNVPLEARMKGVLLRWWQPRHNGTGHDQW 3420
                                   ************************************************************

RELN_SHEEP                         ALDHVEVVLVSTRKQNYMMNFSRQHGLRHFYNRRRRSLRRYP 3460
SP|sp|P78509|RELN_HUMAN|RELN_HUMAN ALDHVEVVLVSTRKQNYMMNFSRQHGLRHFYNRRRRSLRRYP 3460
SP|sp|Q60841|RELN_MOUSE|RELN_MOUSE ALDHVEVVLVSTRKQNYMMNFSRQHGLRHFYNRRRRSLRRYP 3461
SP|sp|P58751|RELN_RAT|RELN_RAT     ALDHVEVVLVSTRKQNYMMNFSRQHGLRHFYNRRRRSLRRYP 3462
                                   ******************************************
```
