# Supplementary material for: Identification of a 31-bp Deletion in the RELN Gene Causing Lissencephaly with Cerebellar Hypoplasia in Sheep
Source: PLoS One. 2013 Nov 19;8(11):e81072. doi: 10.1371/journal.pone.0081072 (PMC3834269; doi:10.1371/journal.pone.0081072)
Supplement: Table S1 — Primers used in the amplification cDNA of the ovine RELN gene. The length of the amplified product and the melting temperature (Tm) of the RT-PCR are also indicated. (DOCX) [file pone.0081072.s002.docx]

**Table S1. Primers used in the amplification cDNA of the ovine *RELN* gene.** The length of the amplified product and the melting temperature (Tm) of the RT-PCR are also indicated.

| **Primer_id** | **Sequence** | **Fragment size** | **Tm** |
| --- | --- | --- | --- |
| RELN_Start1_up | gtgcctccaaaactgaatgag | 399 | 50 |
| RELN_Start1_dn | AGACCCTccaATgCTCTGTG |  |  |
| RELN_start2_up | ACGTTCCGGGACAAGAATAC | 493 | 50 |
| RELN_start2_dn | CCACACTGTTCTCCAGTCTCAC |  |  |
| RELN_start3_up | TCATGTCTGACCACCAGTTTG | 688 | 52 |
| RELN_start3_dn | CCAGCAGGCTTCATACACTTC |  |  |
| RELN_Ex6-11_up | GCTTCTGTCCTTCAGTTTTCC | 688 | 51 |
| RELN_Ex6-11_dn | TTTGCATACAGGGCAACATC |  |  |
| RELN_Ex10-16_up | TGTACCCCTTACCTGGACAC | 630 | 50 |
| RELN_Ex10-16_dn | TTGCAACCATGTCGAGTG |  |  |
| RELN_Ex14-18_up | GAATTCGCTGGAGACAAACG | 687 | 50 |
| RELN_Ex14-18_dn | CCACAAGATTGCTGAAGTCAAG |  |  |
| RELN_EX18-22_up | TACCCCACACATGGACAATC | 396 | 52 |
| RELN_EX18-22_dn | TCCGACATGATCGTGGAAG |  |  |
| RELN_Ex21-25_up | ATTTACATTGGGCAGCAGTG | 623 | 50 |
| RELN_Ex21-25_dn | TTGGATTGACAACTGGGATG |  |  |
| RELN_Ex24-27_up | TTTTCGGGGGAAGACTATGAC | 677 | 50 |
| RELN_Ex24-27_dn | CTGGATGCAAGAGACCTTGG |  |  |
| RELN_Ex27-31_up | AGGCTGTTACCCAGCTTCTG | 676 | 50 |
| RELN_EX27-31_dn | TCCAACTCTCGAAGCAAATG |  |  |
| RELN_Ex28-30_up | CCCGCTGTGGTACAAGATAAC | 579 | 52 |
| RELN_Ex28-30_dn | TCAGCGTAACGAGGTTTTCC |  |  |
| RELN_Ex30-35_up | TCGAATCCAAGGAGGTCAAG | 679 | 52 |
| RELN_EX30-35_dn | ACAGTAGGCTCCACCAAAGC |  |  |
| RELN_EX34-39_up | CCATCAAGTCTGGAACATCG | 600 | 51 |
| RELN_Ex34-39_dn | CAGTTGAGCATCCGACATTG |  |  |
| RELN_Ex38-42_up | GCACCTGAGGAAGATTCAGC | 805 | 52 |
| RELN_Ex38-42_dn | AGAGGCCACCATTGAGAGAA |  |  |
| RELN_Ex41-44_up | CGGAAGTGTGGAATCCTTTC | 691 | 50 |
| RELN_Ex41-44_dn | TGCATTCAACATTGGTAGGC |  |  |
| RELN_EX43-47_up | CGGACATTGCTGTGAATGAG | 655 | 50 |
| RELN_EX43-47_dn | GATCAGGCATCCATACATGAAG |  |  |
| RELN_Ex46-50_up | ATGGCTCTTCATTTCAGTGG | 698 | 50 |
| RELN_Ex46-50_dn | GCACTGAGGAACCAGATAATTC |  |  |
| RELN_Ex49-53_up | AAGGCTGGATCATGCAATTC | 700 | 48 |
| RELN_EX49-53_dn | TGACTGGGATTGATTTCTCG |  |  |
| RELN_EX53-58_up | GGACTTTGCTCCATGAGATG | 819 | 52 |
| RELN_EX53-58_dn | ATAGAGCAGTCGTCGCCTTG |  |  |
| RELN_Ex57-Ex63_up | AGCTGCCTGATCATGTCTCC | 695 | 48 |
| RELN_Ex57-63_dn | AACCTCCACATGGTCCAAAG |  |  |
| RELN_Final_up | ATCACCTGGCATGTCATCG | 499 | 50 |
| RELN_Final_dn | GCACGGATACATCAACATGAAG |  |  |
| RELN_Final_v2_up | TAAATCTCGCACTGCATCTG | 666 | 52 |
| RELN_Final_v2_dn | aaatgggaactcctttgtgg |  |  |
| RELN_gap1_up | CCCCTTCCAATGTCAGTACC | 700 | 52 |
| RELN_gap1_dn | GCAGGAGTCTGAAGATCAGG |  |  |
| RELN_gap2_up | ACACAGCTCAAGACGAGTGG | 580 | 50 |
| RELN_gap2_dn | TAGTCTTCCCCCGAAAACAC |  |  |
| RELN_gap3_up | CAAAGGAGCACCTGAGGAAG | 469 | 50 |
| RELN_gap3_dn | ACAACTCCCGTGTCCGTTAC |  |  |
| RELN_gap4_up | ACGGACACGGGAGTTGTATC | 463 | 52 |
| RELN_gap4_dn | AGGCGAGTAGAAGCAGAACG |  |  |
| RELN_gap5_up | CGGACATTGCTGTGAATGAG | 475 | 52 |
| RELN_gap5_dn | GGGGCCCTATTGAAGTTGTC |  |  |
| RELN_gap6.1_up | CGCTTTCGACATGTTTATGG | 562 | 52 |
| RELN_gap6.1_dn | GCAGTTTGGCCCAGAGTATC |  |  |
| RELN_gap6.2_up | ACCTACCCACTTCCTGAAAGC | 591 | 52 |
| RELN_gap6.2_dn | TGTGTTGGTAAGAGCCTCCTC |  |  |
| RELN_gap6.3_up | GGCGCATTGGTAGTGAGAAC | 630 | 52 |
| RELN_gap6.3_dn | AAGAGGAAGGAAGGCATTGG |  |  |
| RELN_gap6.4_up | ATTGTGGTGGGTTGTGAAGC | 590 | 50 |
| RELN_gap6.4_dn | TTGGTGGCAGCTTGTCTTATC |  |  |
| RELN_gap6.5_up | AAGTTTCCAAGGCGACGAC | 494 | 50 |
| RELN_gap6.5_dn | CATGACCTGTTCCATTGTGG |  |  |
