# Supplementary material for: Identification of a 31-bp Deletion in the RELN Gene Causing Lissencephaly with Cerebellar Hypoplasia in Sheep
Source: PLoS One. 2013 Nov 19;8(11):e81072. doi: 10.1371/journal.pone.0081072 (PMC3834269; doi:10.1371/journal.pone.0081072)
Supplement: Table S2 — Primers used to quantify the levels of RELN mRNA by qRT-PCR. The length of the amplified product and the melting temperature (Tm) are also indicated. (DOCX) [file pone.0081072.s003.docx]

**Table S2. Primers used to quantify the levels of RELN mRNA by qRT-PCR.** The length of the amplified product and the melting temperature (Tm) are also indicated.

| **Primer_id** | **Sequence** | **Fragment size** | **Tm** |
| --- | --- | --- | --- |
| RELN_3repeat_up | ACCTGCATCAAACCAAGAGC | 139 | 60 |
| RELN_3repeat_dn | GTGGAAGGTCGATGGAAATG |  |  |
| RELN_4repeat_up | AGGACGAGGGATTTGTGATG | 204 | 60 |
| RELN_4repeat_dn | TGAGCGATGTTCCAGACTTG |  |  |
| GAPDH_up | TCCTGCACCACCAACTGCTT | 291 | 60 |
| GAPDH_dn | GCAGGTCAGATCCACAACGG |  |  |
